# A Hybrid Hamiltonian for the Accelerated Sampling along Experimental Restraints

**DOI:** 10.3390/ijms20020370

**Published:** 2019-01-16

**Authors:** Emanuel K. Peter, Jiří Černý

**Affiliations:** Institute of Biotechnology of the Czech Academy of Sciences, BIOCEV, Průmyslová 595, 252 50 Vestec, Prague West, Czech Republic; e.peter@fz-juelich.de

**Keywords:** enhanced molecular dynamics simulations, protein folding

## Abstract

In this article, we present an enhanced sampling method based on a hybrid Hamiltonian which combines experimental distance restraints with a bias dependent from multiple path-dependent variables. This simulation method determines the bias-coordinates *on the fly* and does not require *a priori* knowledge about reaction coordinates. The hybrid Hamiltonian accelerates the sampling of proteins, and, combined with experimental distance information, the technique considers the restraints adaptively and in dependency of the system’s intrinsic dynamics. We validate the methodology on the dipole relaxation of two water models and the conformational landscape of dialanine. Using experimental NMR-restraint data, we explore the folding landscape of the TrpCage mini-protein and in a second example apply distance restraints from chemical crosslinking/mass spectrometry experiments for the sampling of the conformation space of the Killer Cell Lectin-like Receptor Subfamily B Member 1A (NKR-P1A). The new methodology has the potential to adaptively introduce experimental restraints without affecting the conformational space of the system along an ergodic trajectory. Since only a limited number of input- and no-order parameters are required for the setup of the simulation, the method is broadly applicable and has the potential to be combined with coarse-graining methods.

## 1. Introduction

Proteins fold into a unique three-dimensional structure after they are translated by the Ribosome. They execute their function through a variety of conformational transitions upon changes in their chemical environment, such as the binding of small molecules, changes in the electrolyte concentration, or the presence of interacting proteins in biological processes [1,2,3]. A number of experimental techniques, namely X-ray crystallography, solution NMR, cryo-electron microscopy, and structural mass-spectrometry have been developed to investigate the structure and the conformational changes of proteins [4,5,6]. Molecular Dynamics simulations (MD) [7] are a valuable tool for complementing the experimental results and to elucidate time-resolved atomistic pictures of configuration spaces of proteins upon mutation or the binding to a specific ligand [8]. Parallelization and the development of efficient algorithms improved the performance of MD by many orders of magnitude [9,10,11,12]. The MD-method largely contributed to the current understanding of protein dynamics and the conformational space in a vast amount of processes. At the same time, enhanced sampling methods, namely accelerated MD [13], Gō-modeling [14,15], Metadynamics [16,17,18,19,20], Monte-Carlo methods [21,22], umbrella sampling, and related techniques such as local elevation [23,24,25,26,27,28], replica exchange MD [29,30,31], replica exchange MD combined with umbrella sampling or other improvements in the sampling [32,33,34,35,36,37,38,39,40,41,42,43,44,45,46], transition path sampling techniques, and statistical sampling methods such as Milestoning [47,48,49,50], accelerated adaptive path-sampling methods [51,52], accelerated methods based on path-integrals [53,54], multiple time-stepping techniques [44,55,56], self consistent field MD approaches [57], enhanced sampling combined with machine learning [58,59,60], and coarse-graining techniques [61,62,63,64,65,66,67,68,69] improved the efficiency of MD by many orders of magnitude. Already at the rise of the MD-method as an important complementary tool, a number of developments have been made to connect the MD-approach with experimental information [70]. A number of early developments used NMR-restraints for the structural determination of proteins from solution NMR-NOESY data [71,72] and NMR-shifts [73], which also has been implemented into enhanced sampling methodologies [74,75,76,77,78]. Alternative restraint techniques have been developed to sample proteins from X-ray small angle scattering experimental data [79,80,81] and cryo-electron microscopy data [82]. A more detailed development evaluated the influence of the applied bias-potential and its impact on the sampling of the underlying NMR-ensemble, which in fact is a time-averaged dataset representing an ensemble of conformations [83,84,85,86,87], which also appeared for biasing methodologies for the simulation of distance-restraints determined in chemical crosslinking experiments combined with mass-spectrometry [87,88,89].

**Figure 1:**
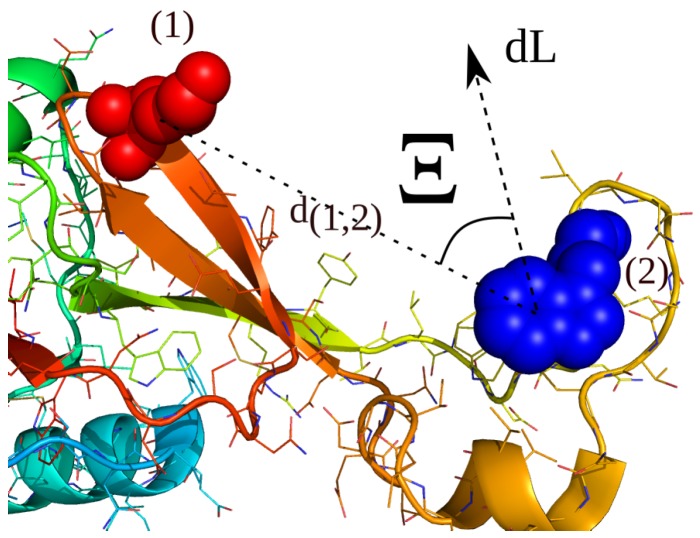
Schematic restraint vector *r*, an instantaneous path-increment dL and the corresponding angle Ξ, which re-weights the adaptive bias coordinate towards an experimental distance information $d_{12}$ between a pair of amino-acids 1 and 2. In this article, we present a method for the coupling of experimental distance restraints along distances between 2 atoms ($d_{12}$) and multiple path-dependent biasing increments ∇H(B). We define a novel restrained biasing Hamiltonian $\nabla H{\left(B\right)}_{R}$ as the overlapping fraction of the path-dependent bias in the dynamical trajectory space with the experimental distance vector. We re-evaluate the un-biased Hamiltonian H(A) using a renormalization technique, which leads to an accelerating hybrid Hamiltonian H(C).

In two recent works, we introduced a renormalization formalism leading to a hybrid Hamiltonian H(C), which introduces additional biasing Hamiltonians H(B) to enhance the sampling in simulations of protein folding and aggregation [90,91]. The novel hybrid Hamiltonian H(C) accelerates the sampling of the system and can shift the resulting statistical averages in the partition function [91]. To bias the simulation along ∇H(B), we developed a technique which uses a history-dependent path-definition *L* resulting in a biased action integral $L_{s}$ and a biased increment $dL_{s}\left(t\right)$ [92]. The enhanced sampling methodology accelerates the sampling of a system, while a minimal set of input parameters, no *a priori* information about reaction pathways or product states is required and the ergodicity of the dynamics is guaranteed. We extend the definition of ∇H(B) to the sampling along multiple biasing increments at multiple time-scales to capture the dynamical heterogeneity of the systems of interest. We then implemented a second adaptive methodology to the restrained sampling along given experimental distance information from NMR-NOESY and chemical crosslinking/mass-spectrometry experiments, which applies the bias indirectly in the form of an overlapping fraction of the biasing increments with the experimental restraint. For a schematic representation see Figure 1. In specific cases, the combination of the adaptive biasing method along pathways *L* and the adaptive reweighting to sampling along given experimental information can achieve a better convergence to the underlying statistical average than the application of restraints in the potential energy space, while a minimal set of parameters *a priori* to the simulation is required. The novel approach also has the advantage that the restrained underlying partition is only dependent on the used parameter set, which is a strong difference to methods, where restraints are applied in the energy space.

We validate the parallel biasing technique on the dipole relaxation of two water models and the conformational landscape of dialanine. As a first application of the novel restrained sampling method, we use the method in folding simulations of TrpCage, where we apply the NMR-data from ref. [93] to sample the conformation ensemble of this protein in explicit and implicit solvent. As a second example, we apply the technique on the conformation ensemble of the Killer Cell Lectin-like Receptor Subfamily B Member 1A (NKR-P1A), where we use restraint information obtained in a combined chemical cross-linking and mass-spectrometry experiment [89]. Because no order parameters are required for the setup of the simulation as in umbrella sampling techniques [94], the restrained path-sampling method is broadly applicable and can also be used in combination with systematic coarse-graining techniques [95,96].

## 2. Results and Discussion

### 2.1. Influence of the Renormalization Parameters on the Time-Dependent Relaxation Behavior in Simulations along Multiple Biasing Increments $dL_{ik}$

We discuss the effect of the multiple renormalized biases and the renormalization parameters $\alpha_{md}$ and $\alpha^{\prime }$ on the dynamical relaxation behavior of a time-dependent quantity *X* describing a system, such as the relaxation of the time-dependent total dipole moment M(t) of a water system. Any quantity X(t) in an unbiased simulation follows a time-correlation function A(X)(t), which can be described by an expansion to *M* monoexponential decay processes with periods $\tau_{m}$, which is defined by the time-behavior of a quantity *X*, rate constants $k_{m}$, and prefactors $A_{m_{0}}$:

(1) $$A\left(X\right)\left(t\right)=\sum_{m}^{M}A_{m_{0}}\exp\left(-k_{m}t\right)\;.$$

**Figure 2:**
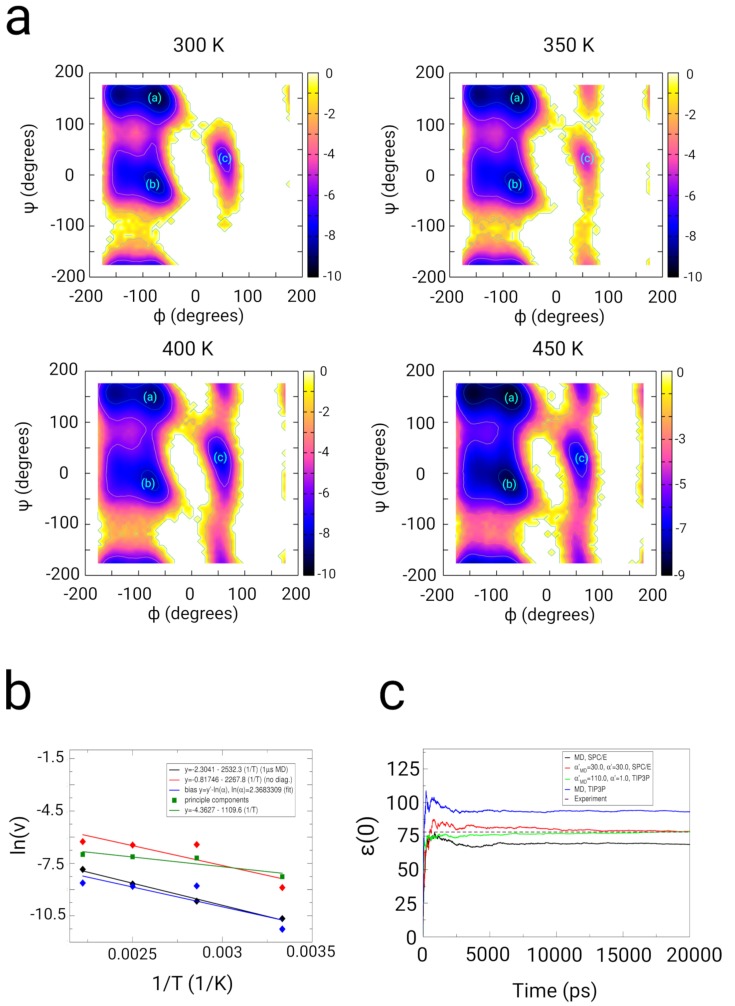
Results from simulations of dialanine peptide in SPC/E water used for the validation of the sampling along $N_{R}$ multiple biasing increments $\nabla H{\left(B\right)}_{ab}$ ($dL_{ab_{ik}}\left(t\right)$, adaptive bias MD) and $\nabla H{\left(B\right)}_{\sigma }$ ($dL_{\sigma_{ik}}\left(L_{ik}\left(t\right)\right)$, path-sampling) to accelerate the sampling, which results in a biased action integral $L_{s}$ and a modification of the un-biased Hamiltonian H(A), which results in a hybrid Hamiltonian H(C). (**a**) Free energy landscapes as function of backbone dihedral angles Φ and Ψ from simulations at the temperatures 300 K, 350 K, 400 K, and 450 K using multipe path sampling in combination with optimization techniques (using principal components of the biasing Hamiltonian H(B)), a number of $N_{R}\;=\;10$ biases, $\beta_{md}^{\prime }\;=\;\beta^{\prime }\;=\;10^{-3}$, $\tau_{1}$ = 0.5 ps, and $\tau_{2}$ = 0.1 ps. Energy values on the color bar are in units of $k_{B}T$. (**b**) Comparison of the logarithm of the transition frequency of the dihedral angle Φ as function of the inverse temperature (300–450 K) in the biased (red and green curve) and 1 μs MD-trajectories (black curve) (blue curve, fit of the biased data to the MD-data for the determination of the linear scaling factor ln(ρ)). In this Kinetic analysis, we determined an acceleration factor ρ equal to 10.68 by which the algorithm accelerates the sampling of dialanine peptide. (**c**) Static permittivities ϵ(0) as function of MD-simulation time using different coupling $\alpha^{\prime }$ and $\alpha_{md}$-parameters in the individual simulations of SPC/E and TIP3P water compared with conventional MD simulations.

In contrast to the equilibrium MD case, a bias coupled to a set of $N_{R}$ biases to *N* atoms, with $\tau_{ik}=\tau_{1_{ik}}$ and $\tau_{ik}=\tau_{2_{ik}}$ leading to an actual acceleration in terms of a change in the time-correlation function. That results in a modified relaxation behavior, affecting all dynamical quantities (that can lead to accelerated folding times, modified diffusion constants, and re-orientation Kinetics of H-bonds or dipoles in the system. There is also an effect on quantities such as the static and dynamic dielectric properties), which we write as an heuristic equation (as described in our simulation results of the dielectic response of SPC/E and TIP3P water):

(2) $$\begin{array}{ccc} A_{m}\left(X\right)\left(t\right) & = & \sum_{m}^{M}\left(A_{i_{0}}^{\prime }\sum_{i}^{N_{R}}A_{im_{0}}^{\prime }\right)\\ & \times & \left(\exp\left(-\left(k_{m}^{\prime }+\sum_{i}^{N_{R}}\;k_{im}\right)t_{rel}\right)\right)\;, \end{array}$$

where $N_{R}$ stands for the number of renormalized biases and $k_{i_{R}}$ is the rate-constant within each bias with index *l*, and we note that the time *t* changes to a relative time $t_{rel}$, which scales linear with an acceleration factor ρ>1: $t_{rel}=\rho t$, as long as the coupling to the underlying un-biased gradient remains sufficiently low ($\beta_{ik_{MD}}^{\prime }=\beta_{ik}^{\prime }\leq 1\times 10^{-4}$, $\eta_{md}^{\prime }=\eta^{\prime }\approx \;25$; see Section: Methods, E: ’Renormalization to the underlying Hamiltonian’) in the enhanced sampling simulation. The relation (2) shows that depending on the magnitude and the parameters ($\beta_{md}^{\prime }$, $\beta^{\prime }$, $\eta^{\prime }$, $\eta_{md}^{\prime }$, $N_{R}$, and the coupling times $\tau_{1}$, $\tau_{2}$), the Hamiltonian of the system and the time-correlation behavior in the simulation are modified, while the processes depending from the parameters *A* and rate constants *k* still are described by modified monoexponential time-dependent decays, since the renormalization and the conditions on *adaptive bias MD* obey the principle of action as described in the Equation (19). That way, dynamical quantities such as dielectric quantities related to dipole fluctuations and in general fluctuation-dependent properties related to a linear response of the system can effectively be varied through the choice of the bias-parameters. Finally, we mention the boundary case of an infinite number of biases $N_{R}$. As stated in the Equation (1), the time-dependent property A(X)(t) will not converge within an imaginary infinite sampling period, if an infinite number of applied biases *s* would be used for this simulation. Thus, the number of applied biases has to be comparatively low in the range from 1 to $N_{R}=20$ biases. We validated the influence of the algorithm in simulations of TIP3P and SPCE/E water, where we measured the impact of the fluctation dependent parameters on the dielectric properties of water in comparison with experiments [97,98,99,100,101] (see supplementary information: Section 2: Water simulations. Supplementary Tables S1 and S2, Figures S1 and S2). Using suitable parameters for $\eta_{md}^{\prime }$ and $\eta^{\prime }$, we successfully could change the dielectric constant of SPC/E (MD: ϵ(0)≈70) and TIP3P water (MD: ϵ(0)≈100) to the value observed in experiments at approximately 78 (see Figure 2c). Using suitable parameters for $\tau_{1}$ (adaptive bias MD), $\tau_{2}$ (path-sampling), $\eta_{md}^{\prime }$ and $\eta^{\prime }$, the dynamic relaxation of the dipole moment of water could also be driven towards the experimental dielectric relaxation spectra.

### 2.2. Dialanine

#### 2.2.1. Results

We used dialanine as a test system to examine the effect of the algorithm on a simple and small peptide. Dialanine has emerged as a prominent system for the validation of enhanced sampling algorithms [16,102,103] and has been used to study the kinetics of transitions along the reactive coordinates [47,104,105] (the dihedral angles Φ, Ψ, and χ). In validation simulations of dialanine, we applied the principal components of the multiple pathways derived from orthogonal vectors $dL_{{⊥}_{ik}}$ to the sampling of dialanine (see Section: Methods). In the simulation at 300 K using the optimized sampling scheme along principal components, we observe an improved partition of the population densities in the partition at $-50^{o}<\Psi <50^{o}$, $-100^{o}<\Phi <0^{o}$ (−8 $k_{B}T$) (minimum (b)), $100^{o}<\Psi <180^{o}$, $-150^{o}<\Phi <0^{o}$ (−9 to −10 $k_{B}T$) (minimum (a)), and the population at $0^{o}<\Psi <100^{o}$, $-50^{o}<\Phi <100^{o}$ (minimum (c)) (see Figure 2a). We compared MD- (1 μs) and adaptive path-sampling simulations (50 ns) (path-sampling simulations without principal components, path-sampling simulations using principal components) at 4 temperatures ranging form 300 to 450 K. We analyzed the transition frequency of the Φ-angle of dialanine as function of time and determined the escape frequency as function of the inverse temperature (see Figure 2b) [90]. In the path sampling simulations without the use of principal components, we find transition times $\tau_{\Phi }$: at T = 300 K $\tau_{\Phi }$ = 7228 ps, at T = 350 K $\tau_{\Phi }$ = 614 ps, T = 400 K $\tau_{\Phi }$ = 634 ps, and at T = 450 K $\tau_{\Phi }$ = 522 ps. The biased simulations using principal components results in transition times $\tau_{\Phi }$: at T = 300 K $\tau_{\Phi }$ = 3873 ps, at T = 350 K $\tau_{\Phi }$ = 1325 ps, T = 400 K $\tau_{\Phi }$ = 1236 ps, and at T = 450 K $\tau_{\Phi }$ = 1083 ps. The MD-results averaged over 1 μs yields transition times at T=300 K $\tau_{\Phi }$ = 42696 ps, T = 350 K $\tau_{\Phi }$ = 15759 ps, T = 400 K $\tau_{\Phi }$ = 5877 ps, and T = 450 K $\tau_{\Phi }$ = 2550 ps [90]. From the linear regression along the 3 data-sets, we determine activation energies $E_{a}$ of 21.05 kJ/mol for MD, $E_{a}$ equal to 18.77 kJ/mol for the biased simulations and $E_{a}$ equal to 9.2 kJ/mol in the biased simulation using the principal components (see Section 3). In the case of the accelerated simulations along principal components, we observe a shift in the transition frequencies at higher temperatures in contrast to standard MD and path-sampling leading to a shift in the value of $E_{a}$. At the same time, we yield an approximately identical partition along the angles Φ and Ψ in the case of the sampling along principal components. From the absolute shift of the transition frequencies, we determined an absolute acceleration factor ln(ρ) for this transition equal to 2.39, leading to an acceleration factor ρ equal to 10.68 by which the biased simulation is faster than equilibrium MD.

#### 2.2.2. Discussion

In our recent work [90], we compared the quality of the sampling using the adaptive path MD, the path-sampling and the hybrid algorithm and found that the partition along the Φ and the Ψ-angle from the 1 μs with the hybrid- and the path-sampling simulation deviated by approximately ≤1$k_{B}T$ in the high energy regions, corresponding to 12.5% in relation to the absolute scale of the free energy difference ΔF. With the extended hybrid sampling methodology based on multiple pathways $dL_{ik}$, the renormalization and the propagation along principle components, we obtain the correct partition in the FEL along Φ and Ψ with approximately identical free energy values. We also obtain quantitatively correct variations in the transition frequencies of the Φ-angle in comparision with 1 μs MD simulations [92].

### 2.3. Simulations with Restraints from NMR NOESY spectra: TrpCage Folding

#### 2.3.1. Results

**Figure 3:**
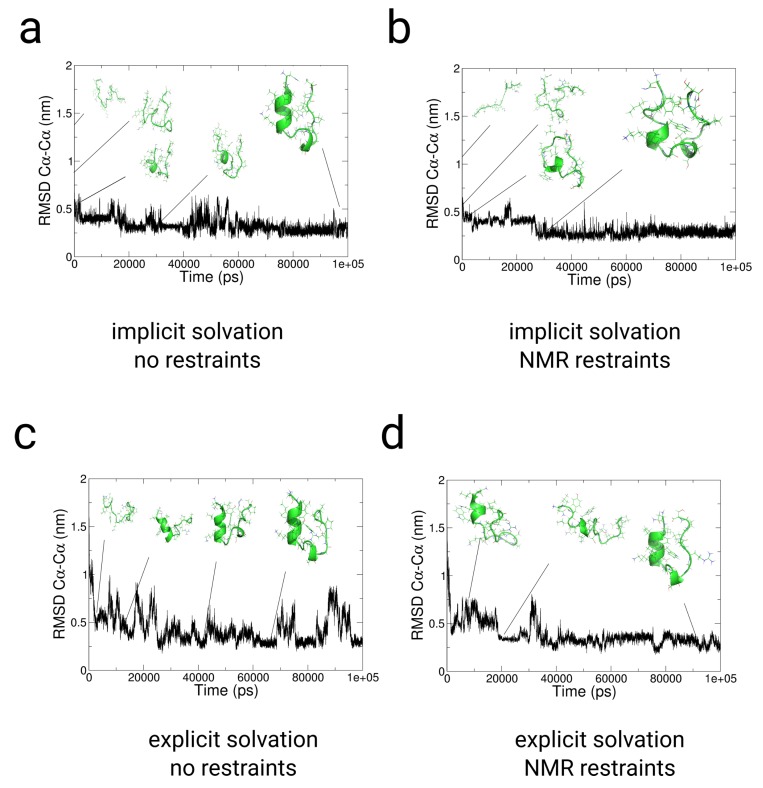
Results from path-sampling simulations with and without restraints from NMR-NOESY measurements of TrpCage starting from an extended conformation [93] in implicit (**a**,**b**) and explicit solvent (**c**,**d**). Panels (**a**,**c**) Results from un-restrained folding simulations of TrpCage in implicit and explicit solvent. Panels (**b**,**d**) Results from folding simulations of TrpCage with NMR restraints in implicit and explicit solvent. (**a**–**d**) $RMSD_{C\alpha -C\alpha }$ to the backbone of NMR-model #1 of PDB: 1l2y. In each of the cases, we observe folding to $RMSD_{C\alpha -C\alpha }$ lower than 0.23 nm. The application of the set of restraints (**b**,**d**) changes the partition of conformations and prevents spontaneous unfolding to configurations at $RMSD_{C\alpha -C\alpha }$ greater than 0.4 nm as we observe in path-sampling simulations without restraints (**a**,**c**).

We used restraints from NMR-NOESY data from ref. [93] for folding simulations of TrpCage in implicit and explicit solvent (See the supplementary information for the restraint data used for the simulations). In the implicit solvent simulations with and without restraints, we observe a fast collapse from the extended conformation to a near native conformation at $RMSD_{C\alpha -C\alpha }\approx$ 0.5 nm within few ps (see Figure 3a,b). The subsequent 2 trajectories show a comparatively fast conversion to the folded state within 20–25 ns to $RMSD_{C\alpha -C\alpha }\;<\;$0.24 nm. The trajectories indicate a relative independence from the choice of the ranges of $\tau_{1}$ and $\tau_{2}$, while lower coupling times lead to a higher propensity for the native structure of TrpCage (see Figure 3b). In the explicit solvent simulation without restraints, the folding-pathway of the peptide is initiated with the formation of a bend in the region from Lys8 to Gly10 at t = 3.8 ns, followed by the formation of a coiled structure at 6.3 ns in which Leu7, Arg16, Asp9, and Pro18 are interacting non-specifically. At a MD-time of 12 ns, we observe the formation of an α-helix between residues Asn1 and Pro12, corresponding to the native N-terminal α-helix. After the process of helix formation, the backbone of the N-terminus between residues 1 and 8 remains at an $RMSD_{\Phi }<20^{o}$, $RMSD_{\Psi }<20^{o}$, which indicates that the structure remains close to the native state within this region already after 12 ns till the end of the simulation (see Figure 3c). At later stages of the simulation, we observe 2 jumps in the $RMSD_{C\alpha -C\alpha }$ from ≈0.36 nm to a value >0.7 nm, followed by a collapse of the peptide to $RMSD_{C\alpha -C\alpha }<0.25$ nm at 23 ns. Within the remaining simulation time, we observe a population of states in the range of $0.2\;<\;RMSD_{C\alpha -C\alpha }\;<\;0.8$ nm, while the system mostly resides between $0.2\;<\;RMSD_{C\alpha -C\alpha }\;<\;0.4$ nm. In contrast to the path-sampling result without restraints, the simulation using NMR-NOESY restraint data in explicit solvent shows a slower decay to a near native conformation within the first 20 ns. During this period, the peptide populates unfolded configurations at $RMSD_{C\alpha -C\alpha }>$ 0.5 nm till a partially folded state is accessed in which Trp6 is exposed to the solvent, and parts of the α- as well as the 3–10 helix are formed (see Figure 3d). The initial population of unfolded states is followed by a re-opening of the peptide, which is expressed by a jump in the $RMSD_{C\alpha -C\alpha }\;>\;$0.5 nm and a collapse to $RMSD_{C\alpha -C\alpha }\;<\;$0.25 nm at approximately 38 ns. In the subsequent trajectory, the protein populates states between 0.3$>RMSD_{C\alpha -C\alpha }\;>\;$0.17 nm.

**Figure 4:**
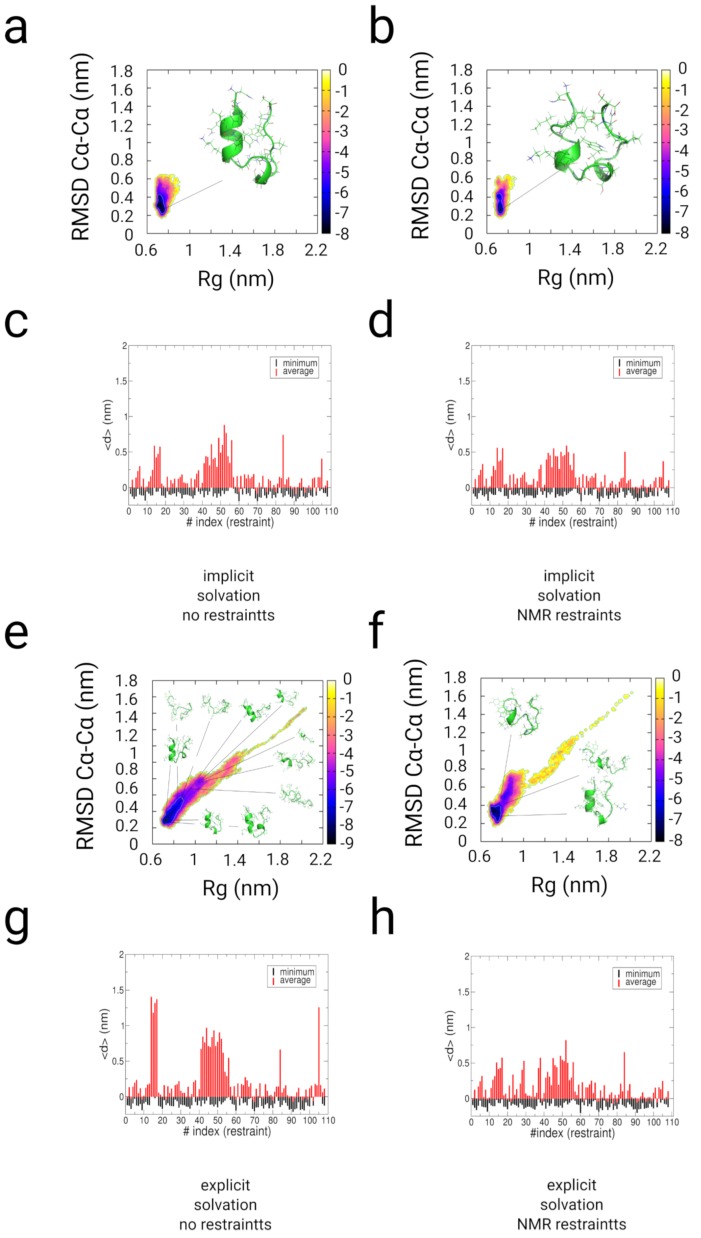
Results from enhanced sampling simulations along multiple pathways with and without restraints from NMR-NOESY experiments on TrpCage. We started the simulation from an extended conformation [93] in implicit (**a**–**d**) and explicit solvent (**e**–**h**). (**a**,**c**,**e**,**g**) Results from path-sampling simulations without restraints in implicit and explicit solvent (using the multiple path sampling methodology without experimental restraints). (**b**,**d**,**f**,**h**) Results from restrained simulations using the experimental NMR restraints for TrpCage. (**a**,**b**,**e**,**f**) Free energy landscapes as function of $RMSD_{C\alpha -C\alpha }$ and radius of gyration (Rg). (**c**,**d**,**g**,**h**) Minimal and average value of the difference of the distance between restraint indexed atoms and the experimental value. The populations in the free energy landscapes of TrpCage are shifted in the restrained simulations with a significantly lower propensity of the unfolded ensemble at $RMSD_{C\alpha -C\alpha }$ greater than 0.4 nm. The presence of the explicit solvent stabilizes near native configurations and leads to a better convergence of the simulation to the set of experimental restraints. Energy values on the color bars are in $k_{B}T$ units.

The free energy landscapes and the cluster population analysis of the implicit and explicit-solvent trajectories of TrpCage indicate that the native state of TrpCage represents the main conformer in the restrained simulations and to a smaller extent in the path-sampling simulation without experimental restraints (see Figures S4–S7 and Figure 4a,b,e,f). In the implicit solvent simulations, the native state is populated with 98.2% and 82.7% ($RMSD_{C\alpha -C\alpha }\;<\;0.25$ nm). In the path-sampling simulation in explicit solvent without restraints, the native state is populated with a propensity of 57.1%, while the restrained simulation yields a population of approximately 80% ($RMSD_{C\alpha -C\alpha }\;<\;0.25$ nm). All simulations populate a native basin at ($RMSD_{C\alpha -C\alpha }<0.25$ nm at free energy values of approximately −8 to −9 $k_{B}T$. We observe the population of states at $RMSD_{C\alpha -C\alpha }>0.4$ nm. In the case of the folding simulation without experimental restraints, the unfolded region is wider and ranges from 1.1>Rg>0.8 nm, while the restrained simulation in explicit solvent populates states at 0.9>Rg>0.8 nm (−6 to −7 $k_{B}T$).

We tested the fulfillment of the distance restraint in the measurement of the difference of the inter-atomic distances of each individual restraint as function of the restraint-index and the experimental values (see Figure 4c,d,g,h and Figure S3). In the distance profiles as function of time and the partitions, we observe values equal to and lower than zero for each of the restraints. We observe larger fluctuations with values larger than 0.5 nm for the restraints between Pro19/Tyr3 (restraint-index: 14–17) and the restraints between Pro12-Pro18/Trp6 (restraint-index: 41–52), which is indicative of a larger flexibility in that range of distances due to several re-openings of the α-helix–Poly-Pro contact in all simulations and the open state in the early stages of the explicit solvent simulations (see Figure 4c,d,g,h). In the explicit solvent simulations, the application of restraints shifts the partition towards a higher degree of the fulfillment of the distance restraints in contrast to the path-sampling simulation where no experimental restraints are applied. The results in the fulfillment of the restraints between Pro12-Pro18/Trp6 (restraint-index: 41–52) and Pro19/Tyr3 (restraint-index: 14–17) indicate that the application of our restrained MD-method improves the sampling of NMR-related configurations by approximately 20–25% in comparison with the folding simulation using the multiple-path sampling method without NMR restraints.

#### 2.3.2. Discussion

The ensemble of pathways of folding of the TrpCage peptide contains a variety of conformational states. Our previous studies on folding of that peptide indicated prior formation of the 3–10 helix initiated by the fast appearance of a turn in the same region [90,92,106,107]. After that first event, we observed that the folding pathways can differ in the formation of first tertiary contacts between the central tryptophan and the poly-proline helix or the first formation of the N-terminal α-helix, which has been identified as a major rate-limiting step in the literature [108,109,110,111,112,113,114]. The sequence of events observed in our previous studies is in agreement with our results, while we do not observe a long-lived molten globule state as part of the folding pathway, which has been reported in two independent works [106,112]. The Kinetically trapped molten globule state is part of the ensemble of folding pathways of TrpCage, while we anticipate that an energy barrier separates that conformation from the set of major pathways of folding. Specific Monte Carlo moves or the choice of a set of biases can guide the system towards that molten globule state [90]. We observe that the application of the NMR-restraints improves the sampling of NMR-related configurations by approximately 20–25% in comparison with the multiple-path sampling simulation without restraints. Our observations indicate that the contact between Trp6/Tyr3 and the Poly-Proline helix is not as stable as indicated in the NMR-structural ensemble (PDB: 1l2y) and open-states as well as partially unfolded conformations might also be part of the actual NMR-ensemble. That result is also underlined by the free energy partitions as well as our analysis of the folding pathways, which are in agreement with previous investigations using different simulation methodologies [90,92,106,107,112,115,116]. From the energy differences of the unfolded state between the implicit and the explicit solvent simulation, we anticipate a stabilization free energy of the unfolded state of TrpCage due to the solvation of approximately 2–4 $k_{B}T$.

### 2.4. Simulations with Restraints from Chemical Crosslinking Data: NKR-P1A

#### 2.4.1. Results

As second application, we simulated NKR-P1A in implicit and explicit solvent using the chemical crosslinking distances and distance-vectors as restraints (see supplementary information, data from ref. [89]). NKR-P1A is an important activating receptor expressed at the surface of natural killer cells [117]. We used the X-ray structure (PDB: 3m9z) as starting structure for the restrained simulations. This structure consists of a central segment of a mixed 3β + 1α + 2β + 1α-fold between residues Leu92/Thr159, Glu185/Tyr215, which are linked via disulfide bridges at Cys122-Cys210, Cys189-Cys202, and Cys94-Cys105. A flexible loop with partial α-helical content is located between Thr159 and Glu185. In a cross-linking experiment and another recent NMR-study, it has been shown that this flexible region is bound to the protein core, while it might still persist as a loop in its secondary structure [89,118]. Another study indicated the role of the loop region as an aggregation interface with interacting proteins [119]. We applied the same set of chemical cross-linking vectors with a target value $d_{0}=0.75$ nm using the crystal structure as a starting configuration. A value for $d_{0}$ equal to 0.75 nm leads to an attractive bias vector for all the chemical restraint values larger than that parameter and aims on the formation of approximate contacts between the 2 indexed aminoacids.

**Figure 5:**
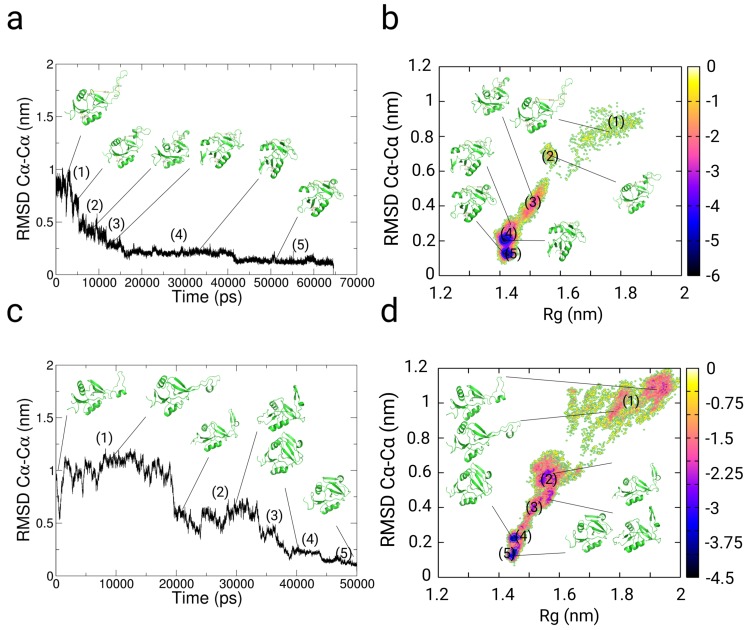
Results from restrained simulations of the Killer Cell Lectin-like Receptor Subfamily B Member 1A (NKR-P1A) along multiple path-dependent biasing variables with chemical cross-linking restraints from ref. [89] (PDB: 3m9z as starting structure). (**a**,**b**) Results from enhanced restrained simulations using the path-dependent biasing approach in implicit solvent. (**c**,**d**) Results from enhanced restrained simulations along multiple path-dependent biases in explicit solvent. (**a**,**c**) $RMSD_{C\alpha -C\alpha }$ to the final structure of each simulation. (**b**,**d**) Free energy landscape as function of $RMSD_{C\alpha -C\alpha }$ and the radius of gyration (Rg). Energy values on the color bar are in units of $k_{B}T$. The protein relaxes quickly out of the extended conformation in both simulations within few ns and adopts 5 different compact states, where the loop region (residues: Arg157-Ser188) are packed to the β-strand interface (residues: Ser188-Asn203) and parts of the second N-terminal helix (residues: Gln135-Ile145) of the monomer.

**Figure 6:**
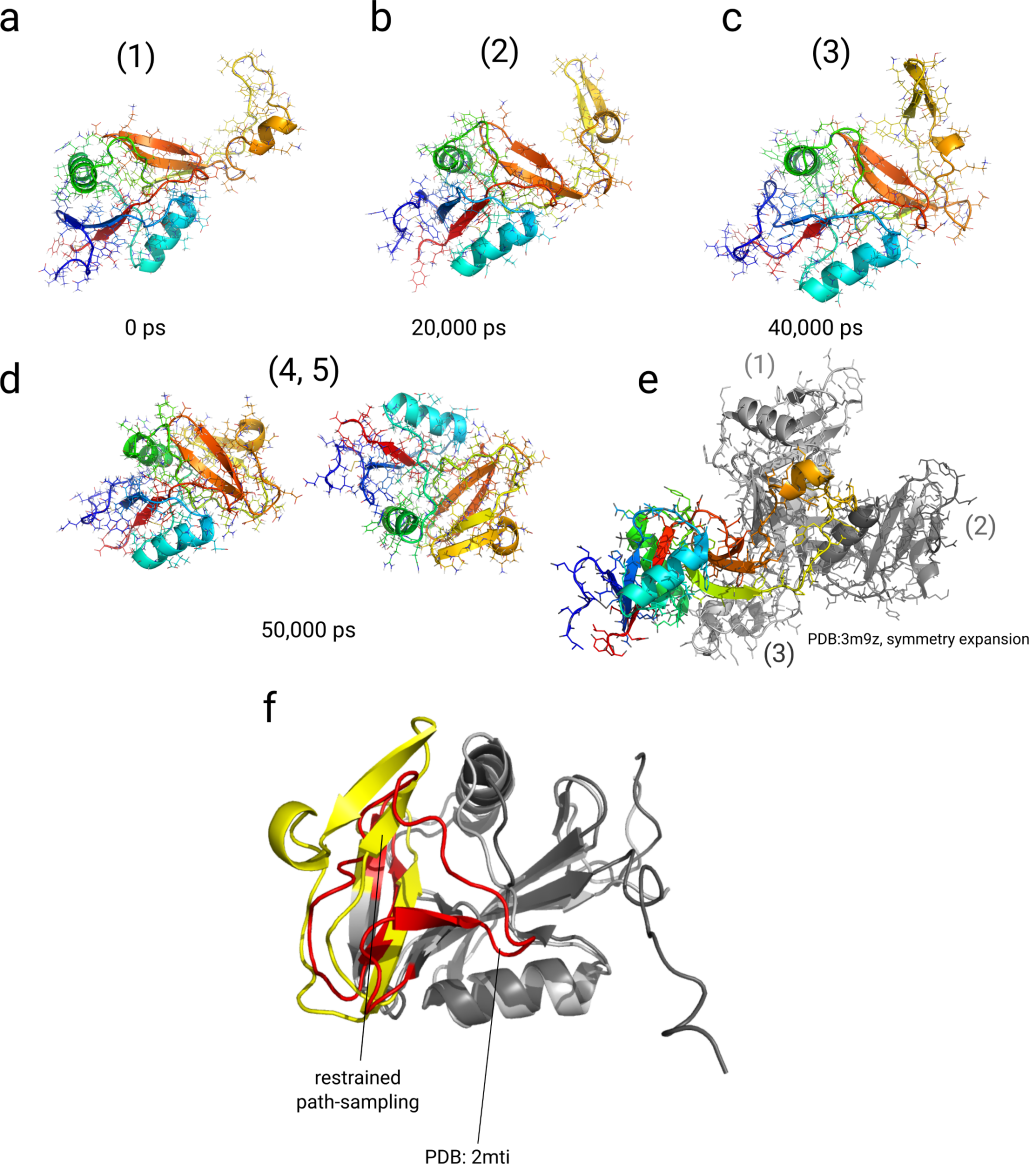
Results from restraint multiple path-sampling simulations of the Killer Cell Lectin-like Receptor Subfamily B Member 1A (NKR-P1A) using chemical cross-linking restraints from ref. [89] in explicit solvent (PDB: 3m9z as starting structure). Conformations at different stages of the restrained simulation (0 ps (**a**), 20,000 ps (**b**), 40,000 ps (**c**), and 50,000 ps (**d**)). (**e**) Symmetry expanded structure of the X-ray structure (PDB: 3m9z) with monomers in contact with the loop region (residues: Arg157-Ser188) of the protein. Monomer (1) (light grey) shares a contact with its 3 β-strands (residues: Tyr149-Ser201) with the loop region in the crystal structure. Monomer (2) (dark grey) is in contact with its second α-helix (Gln135-Lys146) with the loop region. (**f**) Structural overlay of NKR-P1A structure after 50 ns in the explicit restrained simulation (protein core in grey color, loop regions in yellow color) with the experimental NMR-structure (protein core in dark grey color, loop region in red color) (PDB: 2mti [118]). The $RMSD_{C\alpha -C\alpha }$ between the 2 structures equals 0.56 nm.

We simulated the system in implicit and explicit solvent. In the $RMSD_{C\alpha -C\alpha }$ to the final structure of the simulation, we observe a decay from 1 nm to a collapsed state at 0.5 nm within 15 ns in the implicit solvent simulation and within approximately 38 ns in the simulation using explicit solvent (see Figure 5a,c). We then find another drop in the $RMSD_{C\alpha -C\alpha }$ from 0.5 to approximately 0.2–0.3 nm at approximately 42 ns in the implicit solvent simulation and at approximately 44 ns in the simulation in explicit solvent. We analyzed the free energy landscapes as function of the $RMSD_{C\alpha -C\alpha }$ to the final structure and the radius of gyration (Rg) (see Figure 5b,d). In general, we find 5 different states of NKR-P1A throughout the simulation in implicit and explicit solvent. The first state occupies a conformation, in which the loop is flexible and extended. This state is similar to the X-ray structure and populates values in $RMSD_{C\alpha -C\alpha }\;\approx$ 0.8–1.1 nm and Rg ≈ 1.6–1.7 nm in both simulations (see Figure 5a,c and Figure 6a,b). In the explicit solvent simulation, this conformation also includes β-strand formation in one segment of the loop-region between residues Met163 and Asn174 (see Figure 6b). The second state at $RMSD_{C\alpha -C\alpha }\;\approx$ 0.5–0.7 nm and Rg ≈ 1.45–1.65 nm is characterized by a contact between Tyr158, Ile180, and Gly182 at the loop region with residues Phe199, Glu200, and Ser201 at the protein core. At the same time, parts of the loop region rotate around the β-strand axis, while parts of the loop segment are still exposed to the solvent, which is not as strongly visible in the implicit solvent simulation (see Figure 5a,c and Figure 6c). The states (3), (4), and state (5) represent conformations in which the loop region is bound to the protein core at different internal coordinates at the interface with the 2 β-region of the protein core (orange color, see Figure 6c,d). The state (3) is located at $RMSD_{C\alpha -C\alpha }\;\approx$ 0.4 nm and Rg ≈ 1.55 nm, where the loop-region is further rotated around the strand-axis with the same contact-order to the protein-core as in state (2), while residues Asn169, Ile168 at the turn of the strand-region in the loop are closer to the 2β segment of the protein core due to the rotation. In the explicit solvent simulation, the states (4) and (5) are shifted in the Rg-value by 0.025 nm in comparison with the simulation in implicit solvent. In both cases, we observe that both states (4) and (5) contain the strongest level of attraction with energies of −4.5 $k_{B}T$ (-6 $k_{B}T$ in the implicit solvent). In the implicit solvent simulation, the un-bound states (1, 2) are not stabilized as well as in the explicit solvent simulation, leading to a higher free energy of the bound states (3, 4, 5). The states (4, 5) differ in their location along the 2β segment (orange) at the protein core. In general, we observe contacts between Trp165, Trp167 (loop) and Val197 (core), Ile168 (loop) and Leu142 (core), Trp165 (loop) and Phe199 (core). The strand (Asn164-Asn174) within the loop region is stabilized on this segment. In the state (5), the strand reorients and shifts towards one of the α-helical segments (Glu137-Ser144), forming a contact between residues Asn169 (loop) and Gln135 (core) (see Figure 5b,d and Figure 6d).

**Figure 7:**
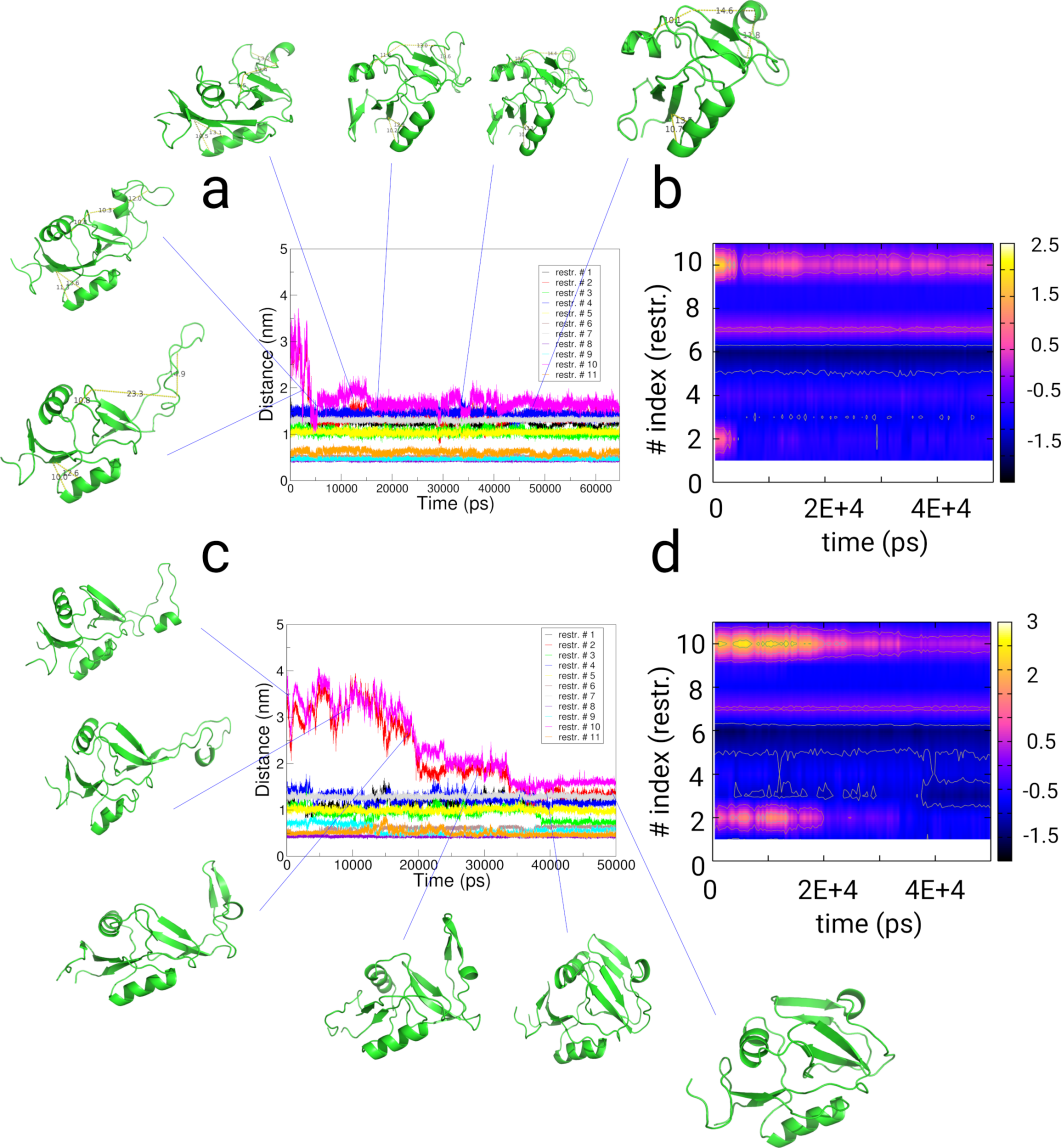
Results from restrained simulations of the Killer Cell Lectin-like Receptor Subfamily B Member 1A (NKR-P1A) using chemical cross-linking restraints from ref. [89] (PDB: 3m9z as starting structure). (**a**,**b**) Results from implicit solvent restrained simulations. (**c**,**d**) Results from restrained simulations in explicit solvent. (**a**,**c**) Distances along 11 distance restraints as function of simulation time given by chemical crosslinking experiments as given in ref. [89]. (**b**,**d**) Difference between the distance and the chemical restraint distance value as given in ref. [89] as function of simulation time and the restraint index. For the restraints #2 and #10, we find conformations, which also populate states with positive values in the difference between the distance and the restraint value.

To summarize, in the measurement of the distances between the restraint-indexed atoms, we observe fulfillment of 9 of the chemical crosslinking distances already at the beginning of the simulation (see Figure S8, and Figure 7a,c). For the restraint-distances with index #2 (residues Lys179-Lys196) and #10 (residues Asp176-Lys196), we observe populations with positive values in the partition of the difference ($d\left(t\right)-d_{0}$(exp)) and the distances over time (see Figure 7). Both restraints are a measure for the orientation of the loop-region to the protein core. The states (1)–(3) populate states with distances larger than 2.0/1.2 nm given as chemical cross-linking distance. The restaints with indexes #1, #3-#6, #8, #9, and #11 populate minima below the chemical cross-linking distance throughout the trajectory, which represent re-orientations within the protein-core. For restraint #7 (Glu147-Lys148), we observe the population of 2 states. Finally, we state that we observe a comparatively low level of fulfillment of the restraint #10 in the time-trace of the simulation, where the difference value converges to a value of approximately 0.7–0.8 nm (see Figure 7b,d and Figure S8).

#### 2.4.2. Discussion

The simulations have relaxed the structures into a conformation in which the loop region is bound to the protein core, and parts of the loop form a β-stranded conformation. In general, we observe the population of 5 different states, while the first state corresponds to the experimental X-ray structure. In the X-ray structure, the open state of the loop-region reflects a bound state to other monomer within the protein crystal, where the loop binds to 2 other monomer units (see Figure 6e). That state might resemble a conformation, in which NKR-P1A binds to other proteins in immune-signaling pathways [119]. The orientation of the loop-region and its partial β-strand secondary structure found in our simulations has a different orientation than in 2 other studies [89,118]. In the 2 structural models, the loop-region is also in contact with the protein core, but it has another orientation as well as a smaller β-strand content. In the 2 modeling studies based on NMR- and chemical restraint data, the loop region binds the protein core in a salt-bridge between Asp176 and Lys196 [89], while an NMR-study indicated a similar packing of that region [118]. Our model differs in the $RMSD_{C\alpha -C\alpha }$ equal to 0.56 nm from the NMR-structure (See Figure 6f) [118]. Using the chemical restraint data, our algorithm reaches a configuration with a potentially better packing of the loop region than the models of the same protein described previously, which underlines the importance of a dynamical sampling along the chemical restraint data. From the energy differences of the unbound state between the implicit and the explicit solvent simulation, we anticipate a stabilization free energy of the open state of NKR1α by the solvent of approximately 2 $k_{B}T$.

## 3. Methods

In a recent work, we introduced a path-variable approach for the sampling along 2 biasing increments derived from the history-dependent momenta *p* and displacements dq [92]. We implemented an adaptive bias MD and a path-sampling component for the accelerated sampling of protein folding and aggregation. In this work, we developed 3 extensions of the previous work: (1) We extended the time-periods for the sampling to the simulation along multiple time-ranges to capture the heterogeneity of structural transitions in biomolecular systems. (2) We implemented a renormalization scheme to facilitate the sampling within the underlying ensemble in dependency of a linear coupling factor. (3) We combined the first 2 methodologies with the simulation along experimental restraint vectors using a novel coupling scheme, in which the accelerating bias along multiple time-ranges is adaptively reweighted. Each of the 3 components are directly linked to each other in a consistent definition of a bias-gradient, while a limited number of input parameters is required. We start with a description of the restraint modeling and compare with the modeling in the energy space. Then we introduce the restrained Hybrid Hamiltonian and the renormalization followed by a description on the impact of the restraint vector on the resulting averages. As a last part, we introduce the enhanced sampling methodology along multiple path-dependent variables and define the bias gradients used in the restrained sampling (see also the Supplementary Material for a derivation of each segment: Section I, Methods).

### 3.1. Theory

#### Adaptive Restraint Modeling

Restraint modeling combined with MD-simulations is a standard tool used for NMR-structure determination, protein structure refinement and applications such as the structural modeling of a system on which restraints are imposed by chemical crosslinking/mass-spectrometry experiments. We note that the restraint modeling strongly depends from the quality of the used experimental data-set, where divergence in the data as for intrinsically disordered proteins would lead to misguided simulations. In the case of a defined experimental dataset containing a detailed residue-based distance information, a very general way to impose the restraint information on the system is the definition of a restraint in the potential energy space *V* of the system, where a classical potential $V_{unbiased}$ is supplemented by an additional restraint potential $V_{r}$ to fulfill an underlying set of experimental information about the system:

(3) $$V=V_{unbiased}+V_{r}\;,$$

leading to forces:

(4) $$\nabla V=\nabla V_{unbiased}+\nabla V_{r}\;,$$

which guides the system towards a potential energy minimum within the given set of restraint parameters, if it is possible for the system to relax into that state. The given set of restraints leads to a faster relaxation of the system towards the potential experimental configurations and the direct comparison of structural information based on experimental input, which directly can connect experiments with simulations on an atomistic length scale. However, an approach imposing direct information on a system containing a system-specific large number of internal degrees of freedom, can guide towards potentially wrong conformations of the system. For example, the novel restrained Hamiltonian can define new local metastable traps in which the system remains for long periods, although such a trap does not correspond to the true experimental ensemble of conformations.

Especially in chemical-crosslinking/mass spectrometry experiments, the experimental information is represented by a distance value $d_{0}$, which corresponds to the geometrical length of the crosslinking molecule between 2 residues. In that case, the restraint between the 2 experimentally crosslinked residues aims on the fulfillment of the condition that the distance *d* between the 2 residues fulfills:

(5) $$d\approx d_{0}\pm \Delta d\;,$$

where Δd represents the structural variability of the inter-residue distance *d* within the ensemble of simulated restrained conformations. That means that the distance information contained in the crosslinking experiment is far from a discrete distance value, but represents an average value $\langle d_{0}\rangle$ with an unknown system specific variation. The same formalism in general holds for NMR-NOESY distance information, where a variability Δd of the simulated conformations around the distance $d_{0}$ also has to be taken into account. Especially, if restraints from chemical crosslinking experiments are used, the variability Δd can be very high, due to the chemical flexibility of the linkers, which motivates our development of an adaptive reweighting of a path-dependent bias in dependency of an angular overlap of the given restraint vector between 2 residues and the adaptive path-dependent bias. That approach has the advantage, that the fulfillment of the restraint corresponding to the equilibrium value $d_{0}$ is not explicitly imposed on the system by a restraint potential $V_{r}$ as function of $d_{0}$, but as function of a new reweighted hybrid Hamiltonian, which only depends from the un-biased forcefield parameter set.

### 3.2. Hybrid Hamiltonian

In the following, we define the unbiased Hamiltonian H(A):

(6) H(A)=T(A)+V(A),

where *T* stands for the Kinetic and *V* is the potential energy. The biasing Hamiltonian H(B) consists of the biased Kinetic T(B) and potential energy terms V(B) in analogy to the definition of H(A). We add that biasing Hamiltonian H(B) to H(A) using a renormalization technique [90], which results in a new Hamiltonian H(C):

(7) $$H\left(C\right)=\frac{H(A)}{1+\alpha_{md}}+\frac{\alpha^{\prime }\left|H\left(A\right)\right|}{\left|H(B)\right|}H\left(B\right)\;,$$

where we distinguish between $\alpha_{md}$ as coupling parameter renormalizing the un-biased Hamiltonian and $\alpha^{\prime }$, which defines the coupling of the bias to the system. We define:

(8) $$\alpha_{md}=\eta_{md}\beta_{md}\left(1-\xi \right)\;,$$

and

(9) $$\alpha^{\prime }=\eta^{\prime }\beta \left(1-\xi \right)\;,$$

where ξ stands for a uniform random number, β, $\beta_{md}$, $\eta^{\prime }$, and $\eta_{md}$ are simulation parameters.

### 3.3. Restrained Hybrid Hamiltonian

The modification of the bias-function to a restrained Hamiltonian $H{\left(B\right)}_{R}$ along experimental restraints leads to a re-definition of the hybrid Hamiltonian to a restrained energy function:

(10) $$H{\left(C\right)}_{R}=\frac{H(A)}{1+\alpha_{md}}+\frac{\alpha^{\prime }\left|H\left(A\right)\right|}{{|H\left(B\right)}_{R}|}H{\left(B\right)}_{R}\;,$$

where the new hybrid Hamiltonian $H{\left(C\right)}_{R}$ consists of a reweighted experimental restraint component *r*. In principle, the total energy remains approximately un-affected, while other properties can be introduced through the bias H(B). We consider a set of distance restraints *r* as an external experimental input for the generation of a biasing energy function $H{\left(B\right)}_{R}$. The energy H(B) consists of an adaptive bias MD component $H{\left(B\right)}_{ab}$ and a path-sampling part $H{\left(B\right)}_{\sigma }$:

(11) $$H\left(B\right)=H{\left(B\right)}_{ab}+H{\left(B\right)}_{\sigma }\;.$$

We then define a restraint bias-gradient $\nabla H{\left(B\right)}_{R}$, which only acts on the system through the cosine of the angle Ξ between the distance-restraint vector and the biasing increments ∇H(B), in a way that the applied bias changes with its orientation relative to the distance vector defining the restraint *r*. Therefor, we calculate the cosine cos(Ξ) between the bias *s* and $q_{12}$ using:

(12) $$\cos\left(\Xi \right)=\frac{\nabla H\left(B\right)\cdot q_{12}}{\left|\nabla H(B)||q\right|}\;,$$

that the resulting bias-gradient changes its sign from $-\frac{1}{2}\pi <\Xi <\frac{1}{2}\pi$ to $\frac{1}{2}\pi <\Xi <\frac{3}{2}\pi$, while the increment in the path remains constant as in the formalism described before. We mention that the cosine fulfills the property that for the angles $\frac{1}{2}\pi <\Xi \leq \pi$ and $\pi \leq \Xi <\frac{3}{2}\pi$ the sign of the applied bias changes towards the direction of the distance restraint. We define the restraint by a distance vector between 2 atoms expressed as $q_{12}=q_{1}-q_{2}$ and modify the applied bias ∇H(B) at time *t* to obtain a restraint bias $\nabla H{\left(B\right)}_{R}$ of the form:

(13) $$\nabla H{\left(B\right)}_{R}=\nabla H\left(B\right)\;\cos\left(\Xi \right)\frac{\left(d_{12}-d_{0}\right)}{|d_{12}-d_{0}|}\;,$$

where $d_{0}$ stands for the equilibrium distance of the restraint (see Figure 1). Equation (10), then, can be expressed as:

(14) $$H{\left(C,\Xi \right)}_{R}=\frac{H(A)}{1+\alpha_{md}}+\frac{\alpha^{\prime }\left|H\left(A\right)\right|}{{|H\left(B,\Xi \right)}_{R}|}H{\left(B,\Xi \right)}_{R}\;.$$

The methodology has the following advantages:

1. The momentum conservation is still obeyed and no additional energy is applied on the system, since the absolute value of ${|\nabla H\left(B\right)}_{R}|$ varies from 0 to |H(B)|, due to 0≤|cos(Ξ)|≤1 for all possible values of Ξ.
2. The effective biasing increments $\nabla H{\left(B\right)}_{ab}$ ($dL_{ab_{ik}}\left(t\right)$, adaptive bias MD) and $\nabla H{\left(B\right)}_{\sigma }$ ($dL_{\sigma_{ik}}\left(L_{ik}\left(t\right)\right)$, path-sampling) are modified for the atoms on which the restraint *r* is applied. That means that the path-increments are changed to $\nabla H{\left(B\right)}_{ab_{R}}$ and $\nabla H{\left(B\right)}_{\sigma_{R}}$ using a flexible bias vector in dependency of the vectorial quantity of ∇H(B) and the restraint *r*, while the absolute increments applied per unit-time remain identical: ${|\nabla H\left(B\right)|=|\nabla H\left(B\right)}_{R}|$.

### 3.4. Restrained Partitions

If a restraint potential $V_{r}$ would be added in conventional sampling along given restraints *r*, the novel restrained partition function $Z_{R}$ is defined by:

(15) $$Z_{R}=\int_{-\infty }^{\infty }\ldots \int_{-\infty }^{\infty }e^{-\frac{1}{k_{B}T}\left(T\left(A\right)+V\left(A\right)+T_{r}+V_{r}\right)}dr^{1}dr^{2}\ldots dr^{N}dp^{1}dp^{2}\ldots dp^{N}\;,$$

where $T_{r}$ is the restrained Kinetic energy. Using our newly developed restrained Hamiltonian $H{\left(C,\Xi \right)}_{R}$, the restrained partition results in:

(16) $$Z_{R}^{\prime }=\int_{-\infty }^{\infty }\ldots \int_{-\infty }^{\infty }e^{-\frac{1}{k_{B}T}\left(H{\left(C,\Xi \right)}_{R}\right)}dr^{1}dr^{2}\ldots dr^{N}dp^{1}dp^{2}\ldots dp^{N}\;,$$

where the hybrid Hamiltonian H(C,Ξ) is defined in the Equation (14), which is a fundamental difference to the partition as described in the first equation, because the scalar quantity of the energy H(C,Ξ) is only affected in dependency of the coupling factors $\alpha_{md}$ and $\alpha^{\prime }$. The shifted average ${\langle X\rangle }_{R}$ through the application of a restraint in the energy space is then written as:

(17) $${\langle X\rangle }_{R}=\frac{Xe^{-\frac{1}{k_{B}T}\left(T\left(A\right)+V\left(A\right)+T_{r}+V_{r}\right)}}{Z_{R}}\;,$$

while the novel sampling methodology using a sampling along reweighted path-dependent biasis is defined as:

(18) $${\langle X\rangle }_{R^{\prime }}=\frac{Xe^{-\frac{1}{k_{B}T}\left(H{\left(C,\Xi \right)}_{R}\right)}}{Z_{R}^{\prime }}\;.$$

The 2 last expressions (17) and (18) also indicate the limitations and advantages of both different restrained sampling techniques: In the first example, where the restraint is applied in the energy space using $V_{r}$, a new potential is added to the system, which can improve the underlying parameter set significantly and leads to an accelerated convergence to a relevant conformation. Examples for applications of that approach can be found in the widely used Gō-modeling approaches [15,120]. However, it might also lead to an ’over-riding’ of the original parameter set V(A), in a way that $V_{r}$ becomes the dominant part in the Hamiltonian. That would mean that essential conformations as part of ${\langle X\rangle }_{R}$ could not be accessed. In the most extreme case, the potential V(A) is ’over-ridden’ by $V_{r}$ that the resulting conformers are not part of the experimental ensemble. In contrast, the approach represented by the Equation (18) has the following limitations and advantages: (1) It requires longer simulation periods for the system to relax into the area of fulfillment of the restraint, since the actual bias is only added in dependency of a necessarily small coupling value α. However, the underlying partition corresponds to the Hamiltonian defined by the forcefield parameters. (2) The resulting average contains primarily only the data in a direct dependency of the used forcefield parameter set due to the definition of the hybrid Hamiltonian. That can result in a limitation of the resulting statistical averages, but has the advantage that the sampled configurations correspond to an approximate equilibrium state.

### 3.5. Path-Sampling Bias: ∇H(B)

#### 3.5.1. Theory

We define the bias Hamiltonian H(B) used for the accelerated sampling along multiple path increments $dL_{ik}$ (for pathways *i* and atom-indices *k*) as well as its modification to its principal components, which is adaptively changed into a bias $H{\left(B\right)}_{R}$ in dependency of a distance restraint *r* given by experimental data. We consider that the simulated system in an equilibrium simulation propagates along a pathway with the general condition that the reduced action *L* as function of momentum *p* and positions *q* remains constant [121,122]:

(19) L=∮pdq=const..

Along a time-dependent MD trajectory of a system exposed to non-zero fluctuations of its momenta dp, we rewrite the Equation (19) as a time-integral:

(20) $$L=\int_{t=t_{0}}^{t_{1}}\left(\frac{d(p(t)dq(t))}{dt}\right)dt^{\prime }=\;const.\;,$$

where $t_{0}$ stands for the start and $t_{1}$ for the end of the simulated MD trajectory. The time-dependent integral is expressed as:

(21) $$L=\int_{t=t_{0}}^{t_{1}}\left(\frac{dp(t)}{dt}dq\left(t\right)+p\left(t\right)\frac{dq(t)}{dt}\right)dt^{\prime }=\;const.\;.$$

We then define a differential dL(t) for a microscopic system, in which fluctuations of the momenta dp(t) occur. That local change in L(t) at the time *t* is defined by:

(22) $$\frac{dL(t)}{dt}=\frac{d}{dt}\left(p\left(t\right)dq\left(t\right)\right)=\frac{dp(t)}{dt}dq\left(t\right)+p\left(t\right)\frac{dq(t)}{dt}\;.$$

We obtain the following differential at time *t*:

(23) dL(t)=p(t)dq(t)+dp(t)dq(t)=(p(t)+dp(t))dq(t).

The expressions in the Equations (20)–(23) relate to the standard MD-case with fluctuations in the momenta dp(t) and displacements dq(t) at times *t*. Any arbitrary biasing technique developed to accelerate a MD simulation adds an instantaneous increment $dL_{s}\left(t\right)$ to dL(t):

(24) $$dL^{\prime }\left(t\right)=dL\left(t\right)+dL_{s}\left(t\right)\;,$$

where we obtain a modified increment $dL^{\prime }\left(t\right)$ and additional changes in the momenta resulting from applied bias-energies affect the instantaneous action of a system in order to reach a faster convergence to the statistical average, i.e., the free energy landscape [16,47,74]. In our recent work, we defined 2 biasing increments $dL_{s}\left(t\right)$: $dL_{ab}\left(t\right)$ (adaptive bias MD) (in the original work, we refer to the variable dL) and $dL_{\sigma }\left(L_{ik}\left(t\right)\right)$ (path-sampling) depending from 2 coupling time intervals $\tau_{1}$ (adaptive bias MD) and $\tau_{2}$ (path-sampling) in which the gradient has been evaluated [92]:

(25) $$dL_{s}\left(t\right)=dL_{ab}\left(t\right)+dL_{\sigma }\left(L_{ik}\left(t\right)\right)\;.$$

We extend the formalism to the sampling within multiple biases defined by the parameter $N_{R}$ standing for the number of multiple biases. We redefine the expression (20) to a multiple sampling in multiple bias-paths along $N_{R}$ multiple biases. The 2 path increments $dL_{ab_{ik}}\left(t\right)$ and $dL_{\sigma_{ik}}\left(L_{ik}\left(t\right)\right)$ are derived from the adaptive bias MD and the path-sampling components of the hybrid algorithm [92]. Both components are added to the un-biased path $\int_{t_{0}}^{t_{1}}dL\left(t\right)dt^{\prime }$ such that the principle of conservation of the action integral is obeyed, and the action integral has to remain constant upon addition of the bias in order to sample the system along an equilibrium trajectory. Taking into account that especially protein systems, and more general aqueous solutions behave heterogeneous related to their relaxation behavior (depending on their actual state), an extension to multiple biases, which is coupled to a finite set of different relaxation times $\tau_{1_{ik}}$ and $\tau_{2_{ik}}$ for $N_{R}$ biases with index *i* and *k* individual atom indices, leads to a more efficient sampling of dynamically heterogeneous systems. That way, we apply that individual number of $N_{R}$ biases within each individual bias *i*, for which a bias *s* is re-evaluated within periods $\tau_{1_{ik}}$ and $\tau_{2_{ik}}$ for the atom with index *k* and is applied over the same period. We define the individual time periods $\tau_{1_{ik}}$ and $\tau_{2_{ik}}$ over $N_{R}$ multiple pathways with the atom-index *k* as:

(26) $$\tau_{1_{i=N_{R},k}}=\sum_{i=1}^{N_{R}}\tau_{1_{k}}\;,$$

and

(27) $$\tau_{2_{i=N_{R},k}}=\sum_{i=1}^{N_{R}}\tau_{2_{k}}\;.$$

Although we apply constant values $\tau_{1}$ and $\tau_{2}$ over all atoms, we introduce the notation with an additional index *k*, which would mean that each individual atom *k* can potentially be coupled to an individual value $\tau_{1_{ik}}$ or $\tau_{2_{ik}}$, which might lead to a higher accuracy to capture individual relaxation times of each atom in the system.

#### 3.5.2. Adaptive Bias MD and Path-Sampling

In the *adaptive bias MD* section of the algorithm we define the first bias component $\nabla H{\left(B\right)}_{ab}$. We introduce $N_{R}$ multiple biases in which the bias is re-evaluated within periods of $\tau_{1_{ik}}$ for the bias with an index *i* and atom *k*. We define the corresponding force $F_{b}\left(t\right)$ at time *t*, and use time-derivative of $\nabla H{\left(B\right)}_{ab}$: $\frac{d}{dt}H{\left(B\right)}_{ab}=\dot{H{\left(B\right)}_{ab}}$. Referring to the previous work, we call that biasing increment $dL_{ab}$. We define the bias along a history-dependent pathway in adaptive bias MD, and introduce a coarsening along multiple finite time increments $\tau_{1_{ik}}$ to coarse-grain the dynamics and to increase the computational efficiency, which leads to an expression for the corresponding force in adaptive bias MD:

(28) $$\begin{array}{ccc} \nabla H{\left(B\right)}_{ab}\left(\tau_{1}\right) & = & \frac{d\dot{H{\left(B\right)}_{ab}}}{d\tau_{1}}d\tau_{1}\\ & = & \sum_{i}^{N_{R}}\sum_{k}^{N}[\gamma_{ik}^{\prime }\left(t\right)\frac{d}{d\tau_{1_{ik}}}\left(\frac{d}{dt}\left[\left(p_{k}\left(t\right)+dp_{k}\left(t\right)\right)dq_{k}\left(t\right)\right]\right)d\tau_{1_{ik}}\\ & + & \frac{d\gamma_{1_{ik}}^{\prime }\left(t\right)}{d\tau_{1_{ik}}}d\tau_{1_{ik}}\left(\frac{d}{dt}\left[\left(p_{k}\left(t\right)+dp_{k}\left(t\right)\right)dq_{k}\left(t\right)\right]\right)]\;, \end{array}$$

where $\gamma_{ik}^{\prime \prime }$ stands for the fluctuation range with the dimension of a length ($\left[nm^{-1}\right]$) and ξ is a normally distributed random number with a weight equal 1 (we used a constant value $\gamma_{ik}^{\prime \prime }=10^{-4}$ in all simulations). For the *path-sampling* component $\nabla H{\left(B\right)}_{\sigma }$, we use a definition of the reactive coordinate $\sigma_{ik}\left(t\right)$ to determine the biasing segment $\nabla H{\left(B\right)}_{\sigma }$ as a function of the increment $dL_{\sigma_{ik}}\left(L_{ik}\right)\left(t\right)$, which we define as [92]:

(29) $$\sigma_{ik}\left(t\right)=dL_{\sigma_{ik}}\left(L_{ik}\right)\left(t\right),$$

A history dependent bias potential $\Phi_{ik}^{t}$ is added in intervals of $\tau_{2_{ik}}$:

(30) $$\Phi_{ik}^{t}=-\frac{\partial }{\partial \sigma_{ik}}W_{ik}\sum_{t\leq t_{b}}\prod_{ik}\exp\left(-\frac{|\sigma_{ik}-\sigma_{ik}^{t-\tau_{2_{ik}}}|}{2\delta \sigma_{ik}^{2}}\right)\;,$$

where the height $W_{i}$ and the width $\delta \sigma_{i}$ are conventionally parameters chosen to provide computational efficiency and an efficent exploration of the free energy $F(\nabla H{\left(B\right)}_{\sigma })$. We define the bias component as:

(31) $$\nabla H{\left(B\right)}_{\sigma }\left(\tau_{2_{ik}}\right)=\nabla_{\sigma_{ik}\left(t\right)}\Phi_{ik}^{t}\;.$$

That formulation constantly drives the system to explore new configurations along the variable $L_{ik}\left(t\right)$ and prevents the system to revisit conformers, which have been sampled previously. *W* and ΔE are constants [123]. In order to accelerate the sampling along H(B) and $H{\left(B\right)}_{R}$, we re-evaluate the principal components of H(B) in order to sample the system along its slowest modes of the 2 segments $H{\left(B\right)}_{ab}$ and $H{\left(B\right)}_{\sigma }$. Therefore, we diagonalize the matrices $dL_{\sigma_{ik}}\left(L_{ik}\right)$ and $dL_{ab_{ik}}\left(t\right)$. The corresponding eigenvectors with the smallest eigenvalue represent the slowest modes and are applied to the system [124].

### 3.6. Bias Gradients

The un-restrained total bias gradient ∇H(B) is then defined as:

(32) $$\nabla H\left(C\right)=\frac{\nabla H(A)}{\left(1+\alpha_{md}\right)}+\nabla H{\left(B\right)}_{ab}\alpha^{\prime }\frac{\left|\nabla H(A)\right|}{{|\nabla H\left(B\right)}_{ab}|}+\nabla H{\left(B\right)}_{\sigma }\alpha^{\prime }\frac{\left|\nabla H(A)\right|}{{|\nabla H\left(B\right)}_{\sigma }|}\;,$$

while the restrained gradient is written as:

(33) $$\begin{array}{ccc} \nabla H{\left(C,\Xi \right)}_{R} & = & \frac{\nabla H(A)}{\left(1+\alpha_{md}\right)}+\left(\nabla H{\left(B\right)}_{ab}\alpha^{\prime }\frac{\left|\nabla H(A)\right|}{{|\nabla H\left(B\right)}_{ab}|}+\nabla H{\left(B\right)}_{\sigma }\alpha^{\prime }\frac{\left|\nabla H(A)\right|}{{|\nabla H\left(B\right)}_{\sigma }|}\right)\\ & \times & \cos\left(\Xi \right)\frac{\left(d_{12}-d_{0}\right)}{|d_{12}-d_{0}|}\;. \end{array}$$

**Algorithm**

- Start loop over MD steps.
- Loop over $N_{R}$ biases, *N* atoms.
- Determine the path-dependent increments $dL_{ik}$ at time *t* for the *adaptive bias MD* segment at periods of $\tau_{1}$ to define the biasing element $\nabla H{\left(B\right)}_{ab}$ (see Equation (28)).
- Evaluate $\Phi_{ik}^{t}$ to increase the history dependent bias at periods of $\tau_{2}$ for the bias with index *i*, atom *k* for the definition of $\nabla H{\left(B\right)}_{\sigma }$ (*path-sampling*) (see Equation (30)).
- Calculate gradients after renormalization in bias with index *i*, atom *k* (path-sampling and adaptive bias MD) (see Equation (7)).
- Determine the principal components of the 2 segments $H{\left(B\right)}_{ab}$ and $H{\left(B\right)}_{\sigma }$.
- Evaluate overlap of bias ∇H(B) with the given restraint to obtain $\nabla H{\left(B\right)}_{R}$ (see Equation (12)).
- Add all gradients.

**List of parameters for the parallel sampling algorithm**

- $N_{R}$, number of parallel biases
- $\tau_{1}$, time-period for the adaptive bias MD for $N_{R}\times \tau_{1_{ik}}$ periods along parallel biases *i* for atoms with index *k*.
- $\tau_{2}$, time-period for the path-sampling for $N_{R}\times \tau_{2_{ik}}$ periods along parallel biases *i* for atoms with index *k*.
- $\gamma_{ik}^{\prime \prime }$, fluctuation-width for the adaptive path-sampling algorithm to determine the deviation of the fluctuating bias around an average value 〈dL〉≈0. We used a constant value $\gamma^{\prime \prime }=10^{-4}$
- *W*, height of the Gaussians applied in the path-sampling. We applied a constant value W=0.1 kJ/mol (ΔE=1000 kJ/mol).
- $\beta_{md}^{\prime }$ and $\beta^{\prime }$, renormalization parameters of the bias to the unbiased gradient (see the definitions of $\alpha_{m}d$ and $\alpha^{\prime }$).
- $\eta_{md}^{\prime }$, fluctuation range of the unbiased gradient (see the definitions of $\alpha_{m}d$ and $\alpha^{\prime }$).
- $\eta^{\prime }$, fluctuation range of the bias related to the unbiased gradient.
- The list of atom-indices per residue are added to a list with a corresponding equilibrium distance $d_{0}$. For the simulations of chemical crosslinking, we added all atom-indices for the first residue and one atom-index for the second residue.

### 3.7. Parameter Selection

In the novel sampling method along multiple time-scales, a system coupled with the parameters $N_{R}=5$, $\tau_{1}=$ 1 ps, $\tau_{2}=$ 2.5 ps is sampled with path dependent biases coupled to the times $\tau_{1_{ik}}=\left\{1.0,2.0,3.0,4.0,5.0\right\}$ ps and $\tau_{2_{ik}}=\left\{2.5,5.0,7.5,10.0,12.5\right\}$ ps. We used characteristic time-periods ranging from 10 ps to 1 ns. The 2 forms of the bias *s* are depending from 2 independent coupling times $\tau_{1_{ik}}$ and $\tau_{2_{ik}}$ ($\tau_{1}$ and $\tau_{2}$ corresponding to $\tau_{1}$ for adaptive bias MD and the period $\tau_{2}$ for the collection of path-sampling coordinates). For the sampling of a small and fast fluctuating system like dialanine with the 2 order parameters represented by its dihedral angles, the choice of the τ parameters has to be sufficiently low to capture the dihedral rotations on the time-scale of less than 1 ps. The true dynamics of a full rotation are slower, but the collection of coordinates facilitating such a transition have to be on the order of less then 10 ps (5–7.5 fs). For the sampling of the folding dynamics of a small peptide, approximately identical parameters can be used. We anticipate that larger systems containing even slower degrees of freedom, such as domain rearrangements need also a larger number $N_{R}$ in the range from 10 to 20. The 2 separate increments $dL_{\sigma_{ik}}\left(L_{ik}\left(t\right)\right)$ and $dL_{ab_{ik}}$ are evaluated within 2 separate modules called adaptive bias MD and path-sampling, which we called hybrid path-sampling algorithm. The magnitude of the added bias ∇H(B) derived from multiple path-dependent increments depends from the coupling parameters $\beta_{md}^{\prime }$, $\beta^{\prime }$ and the fluctuation parameters $\eta_{md}^{\prime }$, $\eta^{\prime }$. Considering the fact that bonded interactions in the biomolecular forcefield can contribute with gradients $>10^{4}$ kJ/mol/nm, a coupling with a factor with a magnitude of $\beta_{md}^{\prime }=\beta^{\prime }=10^{-4}$ corresponds to the order of magnitude of typical non-bonded interactions.

### 3.8. Program and System Preparation

We implemented the method into the GROMACS-4.5.5 code in the routine */src/kernel/md.c* [9]. The distance restaints used for the simulations are given to the program *gmx_mdrun* using a separate parsing routine. We used the AMBER99-SB forcefield for describing the interactions of the proteins and the SPC/E-water model for the explicit solvent simulations [125]. We applied Particle Mesh Ewald electrostatics with a cut-off of 1.0 nm. Van der Waals interactions have been calculated with the same cut-off. We used a time-step of 1.0 fs. The neighborlist has been updated every second time-step. We used the standard generalized Born solvent (GBSA) parameters for the implicit solvent simulations of TrpCage, where electrostatics were calculated using a twin-range cut-off of 1.0/1.2 nm (the long-ranged interaction calculated every second time-step). For the simulations of dialanine, we centered the extended peptide (Ace-Ala-NMe) in a box with dimensions 2.27×2.27×2.27 nm${}^{3}$ and added 371 SPC/E waters. We applied $\tau_{1}=7.5$ ps, $\tau_{2}=5.5$ ps, $N_{R}=30$ and $\beta_{m}d^{\prime }=1\times 10^{-4}$, $\beta^{\prime }=2.5\times 10^{-4}$ ($\eta_{md}^{\prime }=\eta^{\prime }=1.0$). We simulated the system at the 4 temperatures 300, 350, 400, and 450 K. For the explicit solvent simulations of TrpCage, we centered the extended peptide (NLYIQWLKDGGPSSGRPPPS [93]) in a cubic box with dimensions 7.022×7.022×7.022 nm${}^{3}$ and filled the box with 11424 SPC/E waters. We added one chloride ion to the system. For the explicit solvent simulations of the Killer Cell Lectin-like Receptor Subfamily B Member 1A (NKR-P1A), we centered the experimental structure (PDB: 3m9z [117]) in a box with dimensions 6.8260×4.2300×5.0470 nm${}^{3}$ and filled the box with 6990 SPCE/E waters. We added 9 sodium ions to neutralize the system.

For the 2 simulations of TrpCage in implicit solvent we applied $\tau_{1}=$48.5 ps, $\tau_{2}\;=\;$25.5 ps, $\beta_{md}^{\prime }\;=\;\beta^{\prime }\;=\;1\times 10^{-4}$ ($\eta_{md}^{\prime }\;=\;\eta^{\prime }\;=\;1.0$), and $\tau_{1}\;=\;$4.85 ps, $\tau_{2}\;=\;$2.25 ps, $\beta_{md}^{\prime }\;=\;\beta^{\prime }\;=\;1\times 10^{-4}$ ($\eta_{md}^{\prime }\;=\;\eta^{\prime }\;=\;1.0$), and $N_{R}\;=\;5$ multiple biases (we applied the restraint data set from the data-set presented on www.rcsb.org on the TrpCage protein [93]. See the supplementary information, Section 3). For the path-sampling simulation of TrpCage in explicit solvent without restraints, we applied $\tau_{1}\;=\;$2.50 ps, $\tau_{2}\;=\;$1.55 ps, $\beta_{md}^{\prime }\;=\;\beta^{\prime }\;=\;1\times 10^{-4}$ ($\eta_{md}^{\prime }\;=\;\eta^{\prime }\;=\;5.0$), and 5 multiple biases. For the path-sampling simulation of TrpCage in explicit solvent with restraints, we applied $\tau_{1}\;=\;4.85$ ps, $\tau_{2}\;=\;2.55$ ps, $\beta_{md}^{\prime }\;=\;\beta^{\prime }\;=\;1\times 10^{-4}$ ($\eta_{md}^{\prime }\;=\;\eta^{\prime }\;=\;1.0$), and $N_{R}\;=\;5$ multiple biases. For the simulations of the Killer Cell Lectin-like Receptor Subfamily B Member 1A (NKR-P1A) in implicit solvent, we applied $\tau_{1}\;=\;$48.0 ps, $\tau_{2}\;=\;$25.0 ps, $\beta_{md}^{\prime }\;=\;\beta^{\prime }\;=\;1\times 10^{-4}$ ($\eta_{md}^{\prime }\;=\;\eta^{\prime }\;=\;5.0$), and $\tau_{1}\;=\;$4.8 ps, $\tau_{2}\;=\;$2.5 ps, $\beta_{md}^{\prime }\;=\;\beta^{\prime }\;=\;1\times 10^{-4}$ ($\eta_{md}^{\prime }\;=\;\eta^{\prime }\;=\;5.0$), and $N_{R}\;=\;5$ multiple biases (we applied the restraint data set from the data-set presented in ref. [89]. See the supplementary information). For the simulation of the Killer Cell Lectin-like Receptor Subfamily B Member 1A in explicit solvent, we applied $\tau_{1}\;=\;$2.8 ps, $\tau_{2}\;=\;$1.5 ps, $\beta_{md}^{\prime }\;=\;\beta^{\prime }\;=\;10^{-5}$ ($\eta_{md}^{\prime }\;=\;\eta^{\prime }\;=\;1.0$), and $N_{R}\;=\;5$ multiple biases.

**Analysis**

For the analysis of the dielectric behavior of SPC/E and TIP3P water, we refer to the supplementary information. For the cluster analysis of the folding simulations of TrpCage, we used the *gmx_cluster* module using an root-mean square deviation (RMSD) based clustering approach to the backbone of each conformer ($RMSD_{C\alpha -C\alpha }$) with a value of 0.25 nm as criterion for one conformer to belong to one specific cluster [9,126]. We analyzed the transition time of the Φ-angle of Dialanine peptide as the average time $\tau_{a}$ needed to transition between the region $\Phi <0^{o}$ to the region $\Phi >0^{o}$. We then determined the frequency using $\nu_{a}\;=\;1/\tau_{a}$ (see Figure 2b). For the determination of the activation energy $E_{a}$, we used the relations:

(34) $$\nu_{a}=A_{a}exp\left(-\frac{E_{a}}{RT}\right)\;,$$

and

(35) $$\ln\nu_{a}=\ln A_{a}-\frac{E_{a}}{RT}\;.$$

We defined the dihedral Φ by the atoms: C-N-CA-C and Ψ using: N-CA-C-N of the Dialanine peptide. For the measurement of the free energies ΔF, we used:

(36) $$\Delta F=-\;K_{B}T\ln\frac{P}{P_{min}}\;,$$

where $P_{min}$ stands for the minimal probability of the projection on 2 quantities. For the analysis of the fulfillment of a distance restraint, we determined the difference $\Delta =d\left(t\right)-d_{0}$, where d(t) stands for the time-dependent distance and $d_{0}$ for the experimental reference value. We used the Equation (36) for the determination of the free energy differences for the probability to find the system along the reference distance between the actual distance and the experimental reference value. We used the tools *gmx_rms*, *gmx_angle*, and *gmx_mindist* for the calculation of the RMSD to the native structure, the dihedral angles, and the inter-atomic distances. For the determination of the linear acceleration factor ρ, we state that through the renormalization to the underlying Hamiltonian, the transition state for a transition of the system between 2 states remains unaffected, if the coupling factors $\beta_{md}^{\prime }$, $\beta^{\prime }$, $\eta_{md}^{\prime }$, and $\eta^{\prime }$ remain within a range ≪1 [90]. If the transition state energy level ΔE remains approximately unaffected by the applied bias [105,127], the resulting transition time $t_{1,2}$ for a 2-state transition equals:

(37) $$t_{1,2}=\frac{1}{r_{t}}\approx \frac{1}{r_{u}\times r_{c}}\;,$$

where $r_{u}$ stands for the un-biased and $r_{c}$ for the biased rate of the transition. We define a linear acceleration factor ρ which expresses the proportion of the un-biased time $t_{1,2_{u}}$ to the time observed in the accelerated simulation $t_{1,2}$ equals then:

(38) $$\rho =\frac{r_{c}}{r_{u}}\approx \frac{t_{{1,2}_{u}}}{t_{1,2}}\;.$$

## 4. Conclusions

In this article, we presented a methodology which defines a hybrid Hamiltonian H(C), which combines an un-biased Hamiltonian H(A) with a biasing energy function H(B) derived from path-dependent variables. We combine the energy function H(B) with experimental distance restraints *r* to obtain a novel Hamiltonian $H{\left(B\right)}_{R}$. The energy function $H{\left(B\right)}_{R}$ is adaptively reweigted in dependency of the angular overlap between the restraint vector and the experimental distance vector. The biasing function ∇H(B) enhances the sampling along a number of $N_{R}$ intrinsic pathways at multiple time-scales $\tau_{1}$ (adaptive bias MD) and $\tau_{2}$ (path-sampling) in order to capture all possible dynamic modes in the system. We validate the algorithm in simulations of SPC/E and TIP3P water and the conformational landscape of the dialanine dipeptide. We then apply the methodology to the sampling of experimental distance restraints derived from NMR-NOESY data or chemical crosslinking. In simulations of folding of TrpCage using NMR-NOESY experimental restraint data [93], we observe good agreement of the sampled conformation space with the experimental NMR-data. In a second example of restrained sampling of the Killer Cell Lectin-like Receptor Subfamily B Member 1A (NKR-P1A), we apply restraints from chemical crosslinking/mass spectrometry experiments [89]. In both examples, the conformers are in agreement with the applied restraint data. We conclude that the new sampling technique has the advantage of an accelerated sampling of any underlying system, while a minimal amount of *a priori* input is required. The adaptive restraint technique guides the system along experimental restraint vectors in a dynamic way leading to an ergodic sampling within the partition described by the underlying Hamiltonian. Only a small number of input- and no system-dependent order parameters are required for the setup of the simulation as for example in umbrella sampling techniques [94]. The method is broadly applicable and can be used to accelerate the sampling in coarse-grained simulations.

## Supplementary Materials

The following are available online at http://www.mdpi.com/1422-0067/20/2/370/s1. See supplementary material for the simulations, where we investigated the impact of the enhanced sampling simulation parameters on the dielectric behavior of 2 water models and the restraint parameters used in the simulations of TrpCage and NKR-P1A.
