# Supplementary material for: A Hybrid Hamiltonian for the Accelerated Sampling along Experimental Restraints
Source: Int J Mol Sci. 2019 Jan 16;20(2):370. doi: 10.3390/ijms20020370 (PMC6359555; doi:10.3390/ijms20020370)
Supplement: Supplementary file 1 [file ijms-20-00370-s001.pdf]

International Journal of
Molecular Sciences

MDPI

Article

A hybrid Hamiltonian for the accelerated sampling along experimental restraints - Supplementary Material

Emanuel K. Peter † **and Jiří Černý** † *

† Institute of Biotechnology of the Czech Academy of Sciences, BIOCEV, Průmyslová 595, 252 50 Vestec, Prague West, Czech Republic
* Correspondence: jiri.cerny@ibt.cas.cz; Tel.: +420 325 873 738

Received: 17 December 2018; Accepted: 10 January 2019; Published: 16 January 2019

Keywords: Enhanced Molecular Dynamics simulations, protein folding

---

0.1. Hybrid Hamiltonian for the enhanced sampling of protein folding

We define an ideal biasing Hamiltonian $H(B)$ leading to the real underlying partition of the system represented by a free energy landscape (FEL) along suitable restraint vectors and state that the standard forcefield parameter set describes the Hamiltonian $H(A)$ as a reference energy function. Therefore, any property X as a result from the sampling using the Hamiltonian $H(A)$ leads to a corresponding probability P to match a certain value :

$$P_X = f(H(A))\,. \tag{1}$$

An additional bias $H(B)$ in the energy space as conventionally applied in enhanced sampling changes the resulting probability P' to match a defined target property X to :

$$P'_X = f(H(A) + H(B))\,. \tag{2}$$

The additional bias $H(B)$ is applied along collective variables describing the slowest degrees of freedom of the system, as for example in umbrella sampling [1] and related methods such as Metadynamics [2], conformational flooding [3] or local elevation [4]. We recently have introduced a renormalization scheme to solve the problem that the un-biased probability $P(X)$ can be strongly affected by the added bias $H(B)$ [5]. In this scheme, the applied bias is renormalized to the un-biased Hamiltonian $H(A)$ in a way that its magnitude only equals a fraction of the un-biased Hamiltonian $H(A)$ in dependency of two coupling factors α_{md} and α'. Therefor, we introduce a renormalization of the un-biased Hamiltonian by the same factor, which results in a new Hamiltonian $H(C)$:

$$H(C) = \frac{H(A)}{1+\alpha_{md}} + \frac{\alpha'|H(A)|}{|H(B)|}H(B)\,, \tag{3}$$

where we distinguish between α_{md} as coupling parameter renormalizing the un-biased Hamiltonian and α', which defines the coupling of the bias to the system. Alternatively, the restrained Hamiltonian $H(B)_R$ leads to :

$$H(C)_R = \frac{H(A)}{1+\alpha_{md}} + \frac{\alpha'|H(A)|}{|H(B)_R|}H(B)_R\,, \tag{4}$$

where the new hybrid Hamiltonian $H(C)_R$ consists of a reweighted experimental restraint component r. In principle, the total energy remains approximately un-affected, while other properties can be introduced through the bias $H(B)$. In other words, we generate a new probability distribution

$P''(X)$ as a result from adding a particular bias $H(B)$ in dependency of two linear coupling factors α_{md} and α' :

$$P''_X = f\left(\frac{H(A)}{1+\alpha_{md}} + \frac{\alpha'|H(A)|}{|H(B)|}H(B)\right), \tag{5}$$

which also affects the probabilities $P''(X^{\dagger})^{\dagger}$ to find the transition state $X^{\dagger}$, leading to a behavior which can enhance the sampling :

$$P''^{\dagger}_X = f^{\dagger}\left(\frac{H(A)}{1+\alpha_{md}} + \frac{\alpha'|H(A)|}{|H(B)|}H(B)\right). \tag{6}$$

Alternatively, the restrained Hamiltonian $H(B)_R$ leads to :

$$P''_{X_R} = f\left(\frac{H(A)}{1+\alpha_{md}} + \frac{\alpha'|H(A)|}{|H(B)_R|}H(B)_R\right), \tag{7}$$

while the same dependency holds for the probability to find the transition state $X^{\dagger}$. Through this formalism, we introduce another enhancing fragment $H(B)$ or $H(B)_R$ to the original Hamiltonian $H(A)$, without affecting the total energy of the system. As we show later, we generate an accelerating Hamiltonian $H(B)$ or $H(B)_R$ from the parallel path-increments added to the system in the form of a renormalized fluctuation. The orientiation of $H(B)$ is explicitly determined based on a definition of a path-increment and acts on the system through a renormalized fluctuation. That procedure has the advantage that the propagation of the system remains ergodic as long as the coupling factors α_{md} and α' lie within a low range of approximately $< 10^{-4}$ [5]. Considering the fact that bonded interactions in the biomolecular forcefield can contribute with gradients $> 10^4 - 10^5$ kJ/mol/nm, a coupling with a factor with a magnitude of $\alpha_{md} = \alpha' = 10^{-4}$ corresponds to the order of magnitude of typical non-bonded interactions. We mention that we used parameters in the range $\alpha_{md} = \alpha' = 10^{-8}$ for DNA systems using potentials of mean force derived from PDB-data [6].

0.2. Pathway dependent biasing increments : $\nabla H(B)$

We define the bias Hamiltonian $H(B)$ used for the accelerated sampling along multiple path increments dL_{ik} (for pathways i and atom-indices k) as well as its modification to its principal components, which is adaptively changed into a bias $H(B)_R$ in dependency of a distance restraint r given by experimental data. We consider that the simulated system in an equilibrium simulation propagates along a pathway with the general condition that the reduced action L as function of momentum p and positions q remains constant [7,8] :

$$L = \oint pdq = \; const.. \tag{8}$$

Along a time-dependent MD trajectory of a system exposed to non-zero fluctuations of its momenta dp, we rewrite the equation 8 as a time-integral :

$$L = \int_{t=t_0}^{t_1}\left(\frac{d(p(t)dq(t))}{dt}\right)dt' = \; const., \tag{9}$$

where t_0 stands for the start and t_1 for the end of the simulated MD trajectory. The time-dependent integral is expressed as :

$$L = \int_{t=t_0}^{t_1}\left(\frac{dp(t)}{dt}dq(t) + p(t)\frac{dq(t)}{dt}\right)dt' = \; const.. \tag{10}$$

We then define a differential $dL(t)$ for a microscopic system, in which fluctuations of the momenta $dp(t)$ occur. That local change in $L(t)$ at the time t is defined by :

$$\frac{dL(t)}{dt} = \frac{d}{dt}(p(t)dq(t)) = \frac{dp(t)}{dt}dq(t) + p(t)\frac{dq(t)}{dt} . \quad (11)$$

We obtain the following differential at time t :

$$dL(t) = p(t)dq(t) + dp(t)dq(t) = (p(t) + dp(t))dq(t) . \quad (12)$$

The expressions in the equations 9 to 12 relate to the standard MD-case with fluctuations in the momenta $dp(t)$ and displacements $dq(t)$ at times t. Any arbitrary biasing technique developed to accelerate a MD simulation adds an instantaneous increment $dL_s(t)$ to $dL(t)$:

$$dL'(t) = dL(t) + dL_s(t) , \quad (13)$$

where we obtain a modified increment $dL'(t)$ and additional changes in the momenta resulting from applied bias-energies affect the instantaneous action of a system in order to reach a faster convergence to the statistical average, i.e. the free energy landscape [2,9,10]. In our recent work, we used 2 biasing increments $dL_s(t)$: $dL_{ab}(t)$ (adaptive bias MD) (in the original work, we refer to the variable dL) and $dL_\sigma(L_{ik}(t))$ (path-sampling) depending from 2 coupling time intervals τ_1 (adaptive bias MD) and τ_2 (path-sampling) in which the gradient has been evaluated [11] :

$$dL_s(t) = dL_{ab}(t) + dL_\sigma(L_{ik}(t)) . \quad (14)$$

We extend the formalism to the sampling within N_R multiple biases (and optionally N_S multiple simulations) and redefine the expression 9 to a multiple sampling in multiple bias-paths along N_R multiple biases, which the system can undergo simultaneously. The biased simulation then results in the expression of a modified action expression :

$$\begin{aligned} L_m(t) = & \int_{t=t_0}^{t_1} dL(t)dt' + \int_{t=t_0}^{t_1} \sum_i^{N_R} \sum_k^{N} \Big(dL_{ab_{ik}}(t) \\ + & dL_{\sigma_{ik}}(L_{ik}(t)) \Big) dt' = const , \end{aligned} \quad (15)$$

where the multiple biasing pathway dependent increment dL_{s_m} is defined by :

$$dL_{s_m} = \sum_i^{N_R} \sum_k^{N} \Big(dL_{ab_{ik}}(t) + dL_{\sigma_{ik}}(L_{ik}(t)) \Big) , \quad (16)$$

1. Methods

1.1. Renormalization

As given in the equations 3 and 4, we employ a renormalization of the vector $\nabla H(B)$ or $\nabla H(B)_R$ to the underlying ensemble [5], derived from multiple biasing increments $\nabla H(B)_{ab}$ (adaptive bias MD) and $\nabla H(B)_\sigma$ (path-sampling). We apply the relations 3 and 4 for the potential $H(B)$ or $H(B)_R$ acting on an individual atom , where the two parameters α_{md} and α' allow fluctuations of the coupled bias and the un-biased gradient around an average value :

$$\alpha_{md} = \beta'_{md}\eta'_{md} \times (1 - \xi) , \quad (17)$$

and

$$\alpha' = \beta'\eta' \times (1 - \xi) , \quad (18)$$

where η'_{md} and η' define the width of the fluctuation and ξ is a random number $\in]0,1]$. Using the definition :

$$\nabla H(C) = \frac{\nabla H(A)}{1+\alpha_{md}} + \alpha' \frac{|\nabla H(A)|}{|\nabla H(B)|} \nabla H(B)\,, \tag{19}$$

we add the bias $\nabla H(B)$ to the system. As mentioned before, we distinguished between α_{md} to describe the fluctuation of the un-biased MD-gradient and α' for the renormalization of the bias. Using the absolute value of $H(A)$, we can formulate the resulting modified absolute value of the hybrid Hamiltonian $|H(C)|$ of the biased system, which is expressed by :

$$\begin{aligned} |H(C)| &= \frac{1}{1+\alpha_{md}}|H(A)| + \alpha'|H(A)| \\ &= |H(A)|\frac{1+\alpha_{md}+\alpha_{md}^2}{1+\alpha_{md}}\,. \end{aligned} \tag{20}$$

If $\alpha_{md} = \alpha'$ and α_{md} on the order of $0 < \alpha_{md} < 0.1$, then $\frac{1+\alpha_{md}+\alpha_{md}^2}{1+\alpha_{md}} \approx 1$, and we obtain :

$$|H(C)| \approx |H(A)|\,, \tag{21}$$

which means that the energy remains conserved for small α_{md}-values. The renormalization to the underlying Hamiltonian leads to a reduction of the added bias s to the norm of the applied un-biased gradient. We summarize that the magnitude of the added bias s derived from multiple path-dependent increments depends from the coupling parameters β'_{md}, β' and the fluctuation parameters η'_{md}, η'. Considering the fact that bonded interactions in the biomolecular forcefield can contribute with gradients $> 10^4$ kJ/mol/nm, a coupling with a factor with a magnitude of $\beta'_{md} = \beta' = 10^{-4}$ corresponds to the order of magnitude of typical non-bonded interactions. In general, the renormalization procedure guarantees a sampling in which the occurrence of non-equilibrium configurations is prevented and has the advantage that the applied bias enhances the sampling of the system through the addition of renormalized fluctuations. That means that the underlying Hamiltonian is not overdamped through the application of a bias exceeding the un-biased fluctuations, where essential configurations are potentially not accessed. We tested the influence of the α parameters in simulations of SPC/E and TIP3P water, where we investigated the dielectric and the structural properties of water in dependency of the simulation parameters (see Figure 2 b and the supplementary material : Section - 2. Water simulations. supplementary Table 1, Table 2, Figures S1 and S2).

1.2. Adaptive bias MD

For the *adaptive bias MD* section of the algorithm, we derive a history dependent bias $\nabla H(B)_{ab}$ of the form :

$$\nabla H(B)_{ab} = \sum_{i}^{N_R}\sum_{k}^{N} \gamma'_{ik}(t)dL_{ab_{ik}}(t) = \sum_{i}^{N_R}\sum_{k}^{N} \gamma'_{ik}(t)(p_k(t)+dp_k(t))dq_k(t)\,. \tag{22}$$

using a number of N_R-biases in which the bias is re-evaluated within periods of $\tau_{1_{ik}}$ for the bias with an index i and atom k. As we introduced in our previous work, we define the corresponding force $F_b(t)$ at time t, and use time-derivative of $\nabla H(B)_{ab}$: $\frac{d}{dt}H(B)_{ab} = H(\dot{B})_{ab}$:

$$\begin{aligned}\frac{d}{dt}H(B)_{ab} &= H(\dot{B})_{ab}\\ &= \sum_{i}^{N_R}\sum_{k}^{N}\Big[\gamma'_{ik}(t)\frac{d}{dt}\left[(p_k(t)+dp_k(t))dq_k(t)\right]\\ &+ \dot{\gamma}'_{ik}(t)(p_k(t)+dp_k(t))dq_k(t)\Big]\,. \end{aligned} \tag{23}$$

As we defined in the equation 8, the added bias has to fulfill the condition that $\lim_{t\to\infty}\langle dL_{ab}(t)\rangle_t \approx 0$ in order to sample the system at equilibrium. That also implies that the averages of γ_{ik} have to fulfill that $< \gamma'_{ik}(t) >= 0$ and $\left\langle \frac{d\gamma'_{ik}}{dt}\right\rangle \approx 0$. That relation is fulfilled if :

$$\langle \gamma'_{ik}(t)\rangle = 0\,, \tag{24}$$

which is implemented by :

$$\langle \gamma'_{ik}(t)\rangle = \langle \gamma''_{ik} - \xi\gamma''_{ik}\rangle = 0\,, \tag{25}$$

where γ''_{ik} stands for the fluctuation range with the dimension of a length ($[nm^{-1}]$) and ξ is a normally distributed random number with a weight equal 1. We used a constant value $\gamma''_{ik} = 10^{-4}$ in all simulations. To enhance sampling along a history-dependent pathway in adaptive bias MD, we employ a coarsening expressed by :

$$\begin{aligned}\frac{d}{d\tau_{1_{ik}}}H(\dot{B})_{ab} &= \\ &= \sum_{i}^{N_R}\sum_{k}^{N}\frac{d}{d\tau_{1_{ik}}}\times\Big((\gamma'_{ik}(t)\frac{d}{dt}\left[(p_k(t)+dp_k(t))dq_k(t)\right]\\ &+ \dot{\gamma}'_{ik}(t)(p_k(t)+dp_k(t))dq_k(t))\Big). \end{aligned} \tag{26}$$

By taking into account that $\frac{d}{d\tau_{1_{ik}}}\left(\dot{\gamma}'_{ik}(t)(p_k(t)+dp_k(t))dq_k(t))\right) \approx 0$ we use that formalism to define the differential over a finite time increments $\tau_{1_{ik}}$ to coarse-grain the dynamics and to increase the computational efficiency, which leads to an expression for the corresponding force in adaptive bias MD :

$$\begin{aligned}\nabla H(B)_{ab}(\tau_1) &= \frac{dH(\dot{B})_{ab}}{d\tau_1}d\tau_1\\ &= \sum_{i}^{N_R}\sum_{k}^{N}\Big[\gamma'_{ik}(t)\frac{d}{d\tau_{1_{ik}}}\left(\frac{d}{dt}\left[(p_k(t)+dp_k(t))\,dq_k(t)\right]\right)d\tau_{1_{ik}}\\ &+ \frac{d\gamma'_{1_{ik}}(t)}{d\tau_{1_{ik}}}d\tau_{1_{ik}}\left(\frac{d}{dt}\left[(p_k(t)+dp_k(t))\,dq_k(t)\right]\right)\Big]\,. \end{aligned} \tag{27}$$

1.3. *Path sampling*

In the *path-sampling*, we use a definition of the reactive coordinate $\sigma_{ik}(t)$ to determine the biasing segment $\nabla H(B)_\sigma$ as function of the increment $dL_{\sigma_{ik}}(L_{ik})(t)$, which we define as [11] :

$$\sigma_{ik}(t) = dL_{\sigma_{ik}}(L_{ik})(t), \tag{28}$$

with $L_{ik}(t) = \int_{t_0}^{t} \left(\frac{dp(t)}{dt} dq(t) + p(t) \frac{dq(t)}{dt} \right) dt'$, equal to the path integral reached by integration till time t for the bias with index i and atom k. In cartesian coordinates : $\sigma_{ik}(t) = \{L_{x_{ik}}(t), L_{y_{ik}}(t), L_{z_{ik}}(t)\}$ and $L(t) = \{\oint p_{x_{ik}} dx_{ik}, \oint p_{y_{ik}} dy_{ik}, \oint p_{z_{ik}} dz_{ik}\}$. Along $\sigma_{ik}(t)$ a history dependent bias potential Φ^t_{ik} is added in intervals of $\tau_{2_{ik}}$:

$$\Phi^t_{ik} = -\frac{\partial}{\partial \sigma_{ik}} W_{ik} \sum_{t \leq t_b} \prod_{ik} \exp\left(-\frac{|\sigma_{ik} - \sigma_{ik}^{t-\tau_{2_{ik}}}|}{2\delta\sigma^2_{ik}} \right), \tag{29}$$

where the height W_i and the width $\delta\sigma_i$ are conventionally parameters chosen to provide computational efficiency and an efficent exploration of the free energy $\mathcal{F}(\nabla H(B)_\sigma)$. We define the bias component as :

$$\nabla H(B)_\sigma(\tau_{2_{ik}}) = \nabla_{\sigma_{ik}(t)} \Phi^t_{ik}\,. \tag{30}$$

That formulation constantly drives the system to explore new configurations along the variable $L_{ik}(t)$ and prevents the system to revisit conformers, which have been sampled previously. In the implementation for an efficient exploration of this space, we note that our algorithm uses the definition [2] :

$$\delta\sigma_{ik} = |\sigma_{ik}^{t_{b_{ik}}} - \sigma_{ik}^{t'_{b_{ik}}}|\,, \tag{31}$$

where the times $t_{b_{ik}}$ and $t'_{b_{ik}}$ are defined by $\tau_{2_{ik}} = t_{b_{ik}} - t'_{b_{ik}}$. We apply a variable height of each Gaussian added to an individual variable :

$$W_{ik} = W \exp\left(-\Phi^t_{ik}/\Delta E\right) \times \frac{|\sigma_{ik}^{t_{b_{ik}}} - \sigma_{ik}^{t'_{b_{ik}}}|}{\sigma_{ik}^{t_{b_{ik}}}}\,, \tag{32}$$

where W and ΔE are constants [12] We applied constant values $W = 0.1$ kJ/mol ($\Delta E = 1000$ kJ/mol) in all simulations. In order to accelerate the sampling along $H(B)$ and $H(B)_R$, we re-evaluate the principal components of $H(B)$ in order to sample the system along its slowest modes of the 2 segments $H(B)_{ab}$ and $H(B)_\sigma$. Therefore, we diagonalize the matrices $dL_{\sigma_{ik}}(L_{ik})$ and $dL_{ab_{ik}}(t)$. The corresponding eigenvectors with the smallest eigenvalue represent the slowest modes and are applied to the system [13].

2. Results : Water simulations

In this section, we discuss our results from the simulations of 2 water models : TIP3P and SPC/E, with the sampling algorithm along multiple pathways. We tested the influence of the 2 renormalization parameters β, η' and η_{MD} on the time-dependent relaxation of the total dipole moment of the system. We also varied the parameters N_R and the 2 time-periods τ_1 and τ_2 and tested their influence on the dielectric properties of the 2 water models. As key quantities, we chose the radial distribution function $g(r)$, the dielectric permittivities (static) $\epsilon(0)$ (frequency dependent) $\epsilon(\omega)$ and the diffusion coefficients as a quantitative measure of the properties of water in dependency of the simulation parameters.

As stated in the main text, we consider the effect of the multiple renormalized biases and the renormalization parameters on the dynamical relaxation behavior of a time-dependent quantity X describing a system, such as the relaxation of the time-dependent total dipole moment $\mathbf{M}(t)$ of a water system. Any quantity $X(t)$ in an unbiased simulation follows a time-correlation function $A(X)(t)$, which can be described by an expansion to M monoexponential decay processes with periods τ_m, which is defined by the time-behavior of a quantity X, rate constants k_m and prefactors A_{m_0} :

$$A(X)(t) = \sum_{m}^{M} A_{m_0} \exp(-k_m t) \,. \tag{33}$$

In contrast to the equilibrium MD case, a bias coupled to a set of N_R biases to N atoms, with τ_1 and τ_2 leading to an actual acceleration in terms of a change in the time-correlation function. That results in a modified relaxation behavior, affecting all dynamical quantities (That can lead to accelerated folding times, modified diffusion constants and re-orientation kinetics of H-bonds or dipoles in the system. There is also an effect on quantities such as the static and dynamic dielectric properties), which we write as an heuristic equation (as described in our simulation results of the dielectic response of SPC/E and TIP3P water) :

$$\begin{aligned} A_m(X)(t) &= \sum_{m}^{M}\left(A'_{i_0}\sum_{i}^{N_R}A'_{im_0}\right) \\ &\left(\exp\left(-\left(k'_m+\sum_{i}^{N_R}k_{im}\right)t_{rel}\right)\right) , \end{aligned} \tag{34}$$

where N_R stands for the number of renormalized biases and k_{i_R} is the rate-constant within each bias with index l, and we note that the time t changes to a relative time t_{rel}, which scales linear with an acceleration factor $\rho > 1$: $t_{rel} = \rho t$, as long as the coupling to the underlying un-biased gradient remains sufficiently low ($\beta_{ik} \leq 1 \times 10^{-4}$, $\eta' = \eta_{MD} \approx$ 25 - see Section : Methods, E: 'Renormalization to the underlying Hamiltonian') in the enhanced sampling simulation. The relation 34 shows that depending on the magnitude and the parameters (β, N_R and the coupling times τ_1, τ_2), the Hamiltonian of the system and the time-correlation behavior in the simulation are modified, while the processes depending from the parameters A and rate constants k still are described by modified monoexponential time-dependent decays, since the renormalization and the conditions on *adaptive bias MD* obey the principle of action as described in the equation 5 in the main text. In other words, dynamical quantities such as dielectric quantities related to dipole fluctuations and in general fluctuation-dependent properties related to a linear response of the system can effectively be varied through the choice of the bias-parameters.

2.1. Water simulations : System preparation and analysis

We used SPC/E and TIP3P water simulations in order to test the influence of the new simulation method on the correlation behavior of the system and to validate the property of the algorithm to change dynamic properties while structural properties of water remain approximately unaffected.

For the sampling of dielectric properties of SPC/E and TIP3P water, we filled a box with dimensions $1 \times 1 \times 1$ nm^3 with 33 water molecules (SPC/E or TIP3P). We applied PME electrostatics with a cut-off of 0.4 nm, while van der Waals interactions have been calculated using a shift function with the same cutoff. We checked the size-dependency of our results in simulations of a system with size of $5 \times 5 \times 5$ nm^3 filled with 4124 water molecules (PME electrostatics with a real-space cut-off of 0.8 nm, and a cut-off for the Lennard-Jones 12-6 interactions of 0.6 nm). We used the same system and applied identical parameters to test replica exchange MD (REMD) using 4 replicas at 300 K with an exchange interval every 1,000 time-steps. For the REMD simulations, we omitted the determination of frequency dependent dielectric properties. All simulations have been carried out in the *NVT-ensemble* using the Nosé-Hoover thermostat (i.e. the Nosé-Hoover equations of motion) with a coupling interval of $\tau_c = 1$ ps and a reference temperature of 300 K. The small water-systems have been propagated over 20 ns, while the larger reference systems have been sampled over approximately 5 ns.

For the biased water-simulations, we used a β coupling parameter of 5×10^{-4}, 20 replicas and τ_1=5 ps, τ_2=10 ps. The variational parameters have been chosen with a width that no variation of β

and τ_1, τ_2 has been performed. The gradient optimization have been switched off. We systematically varied η_{MD} and η' from 0 to 500.0. For the calculation of frequency dependent properties of the dielectric constant $\epsilon(\omega)$, we calculated the autocorrelation function using the *g_dipoles* module. We normalized the faster decay of the dipole-autocorrelation function from the biased simulations to the decay in the MD-simulations of SPC/E and TIP3P water using the relative positions of the first zero-value of the autocorrelation functions. In other words, we determined the acceleration factor η' of the biased-simulation relative to the un-biased simulation in terms of the decay of the dipole autocorrelation function from 1 to 0 (see Figure 1S), which means that the effective acceleration in the reorientation of the water dipoles introduced by the bias is considered as general acceleration η' in the sampling of the underying system. That prodedure leads to the correct frequency dependent dielectric permittivity in comparison with the experiment (see section : Results and Discussion, Water simulations, Dielectric properties).

The static dielectric constant $\epsilon(0)$ have been calculated using the Kirkwood factor g_k as implemented in the gromacs-4.5.5 analysis module *g_dipoles*, using [14] :

$$g_k = \frac{1}{N\mu^2}(\langle \mathbf{M}^2 \rangle - \langle \mathbf{M} \rangle^2) \,, \tag{35}$$

and

$$\epsilon(0) = 1 + \frac{4}{3}\pi N g_k \mu^2/(k_B T) \,. \tag{36}$$

The frequency dependent dielectric constant $\epsilon(\omega)$ has been calculated using the *g_dielectric* module from GROMACS [15]. That formalism uses a normalized autocorrelation function $\Phi(t)$:

$$\Phi(t) = \frac{\langle \mathbf{M}(0)\mathbf{M}(t) \rangle}{\langle \mathbf{M}^2 \rangle} \,, \tag{37}$$

and uses the Fourier-Laplace transform[16] :

$$\frac{\epsilon(\omega) - 1}{\epsilon(0) - 1} \frac{2\epsilon_{rf} + \epsilon(0)}{2\epsilon_{rf} + \epsilon(\omega)} = \int_0^\infty \left(-\frac{d\Phi(t)}{dt} \right) e^{-i\omega t} dt \,. \tag{38}$$

For the case of the sampling along N_R multiple biases, we refer to equation 34 and state that the static permittivity can be modified in dependency of the coupling parameters η', β and the number of multiple biases N_R. For the 2 processes as described in the equations 35 and 38, the correlation functions then effectively yield different permittivity values. As starting parameters for the fit to the decay of the autocorrelation function, we used a double exponential function with the fit parameters $A = 0.5$, τ_1=10 ps, τ_2=1 ps, $\epsilon(0)$ resulting from the first analysis using the Kirkwood factor, ϵ_{RF}=78.5 and a smoothing over 10 data points.

2.2. Water simulations : Results

2.2.1. Dielectric properties

We determined the static permittivities $\epsilon(0)$ ($\epsilon(\omega)$ at frequency $\omega = 0$) as function of simulation time in biased and unbiased simulations for SPC/E and TIP3P water. Through the investigation of the dependency from the η_{MD} and the η'-parameters on the static permittivity, we determined the property of our algorithm to influence the correlation behavior of the underlying system. We used a static amount of bias-replicas and a constant coupling constant β in order to measure the dependency from the fluctuation dependent parameters η_{MD} and η'. Our results are summarized in Table 2.

In the biased simulations of SPC/E water, a parameter of η_{MD}=500, η'=1.0 yielded the lowest value of $\epsilon(0)$=53. We reach a maximum value in the static permittivity of $\epsilon(0)$=119 using parameters η_{MD}=100.0, η'=100.0, and obtain a permittivity value of $\epsilon(0)$=78.4 using the parameters η_{MD}=30.0,

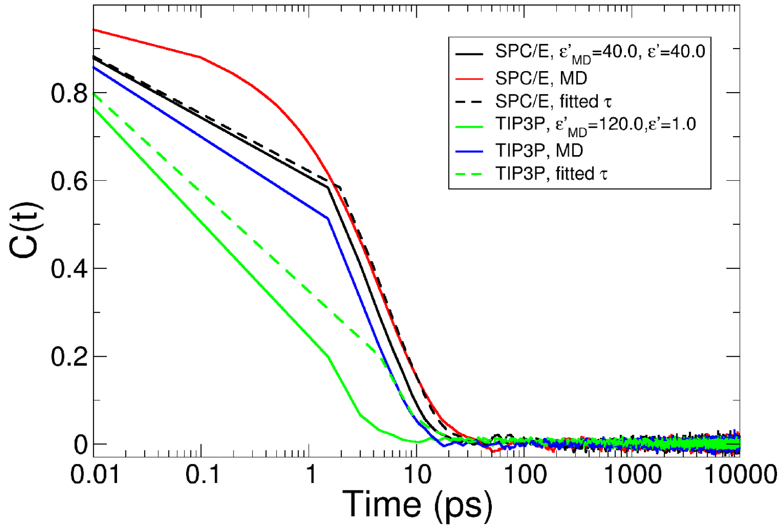

Fig. S1. Normalized dipole autocorrelation functions from MD- and biased simulations of SPC/E and TIP3P water. We scaled the decay time of the autocorrelation result of biased SPC/E and TIP3P water to determine the linear acceleration factor η' by which the reorientation of the dipole of water is accelerated (see section Methods in the main text). We find that η'=10 yields an optimal fit to the decay of the unbiased simulation and also leads to a good agreement with the experimental frequency dependent permittivity $\epsilon(\omega)$ (see main text).

η'=30.0 independent from the dimension of the system (1 nm^3 and 125 nm^3). In the MD-simulations of the systems with dimensions $1 \times 1 \times 1$ nm^3 and $5 \times 5 \times 5$ nm^3, we determine values for the permittivity of $\epsilon(0)$=68.7 and $\epsilon(0)$=66.5 (see Figure 2S c). We mention that the results contain an associated error of approximately $\Delta\epsilon(0) \approx$ 1-2.

Using biased simulations of the TIP3P-water model in systems with the same dimensions, we obtain the lowest value for the static permittivity of $\epsilon(0)$=55 using η_{MD}=500, η'=1.0 . In contrast, we measure a maximum value in the static permittivity of $\epsilon(0)$=92 with the parameters η_{MD}=1.0, η'=1.0. We obtain a permittivity value of $\epsilon(0)$=78.0 using η_{MD}=110.0, η'=1.0. In the MD-simulations of the $1 \times 1 \times 1$ nm^3 and the $5 \times 5 \times 5$ nm^3 box we determine values for the permittivity of $\epsilon(0)$=93 and $\epsilon(0)$=102 (see Figure 2S d). We yielded approximately an identical value in a $5 \times 5 \times 5$ nm^3 box with the same parameters.

We analyzed the frequency dependent permittivities $\epsilon(\omega)$, and observed a decay in $\epsilon'(\omega)_{spec}$ from a value of 78.4 to 2.69 within the frequency range from 8E-3 to 1 THz in the simulation of SPC/E water using η_{MD}=30.0, η'=30.0. The same function decays 4 times slower in the simulation of TIP3P-water using η_{MD}=110.0, η'=1.0 in the frequency range from 8E-3 to 4 THz. In the unbiased MD-simulation of SPC/E water, we observe a decay of $\epsilon'(\omega)_{spec}$ from 72 to 3.2 in the frequency range from 2.5E-3 to 7.5E-2 THz, while we measure a decay from 92.6 to 3.1 within the frequency range from 8.7E-3 to 4 THz in the unbiased MD-simulation of TIP3P-water. In the case of $\epsilon''(\omega)$ (i.e. the imaginary part of the frequency dependent permittivity), we observe a strong effect of the bias on the location of the maximum along the frequency axis. In the simulation of SPC/E water using η_{MD}=30.0, η'=30.0, we find a maximum value of $\epsilon''(\omega)_{spec}$=39.7 at 0.11 THz. For TIP3P water simulated with the parameters η_{MD}=110.0, η'=1.0, we find a maximum value of $\epsilon''(\omega)_{spec}$=31.47 at 0.107 THz. The experimental maximum is located at approximately $\epsilon''(\omega)_{spec}$=37.24 and ϵ=0.12 THz [17]. In contrast, in the unbiased MD-simulations of SPC/E and TIP3P-water, we obtain maxima in $\epsilon''(\omega)_{spec}$=39.69 at ω=0.031 THz (SPC/E) and $\epsilon''(\omega)_{spec}$=44.5 at ω=0.19 THz (TIP3P). In the analysis of the REMD simulations, we omitted this analysis for the REMD trajectories of SPC/E and TIP3P water.

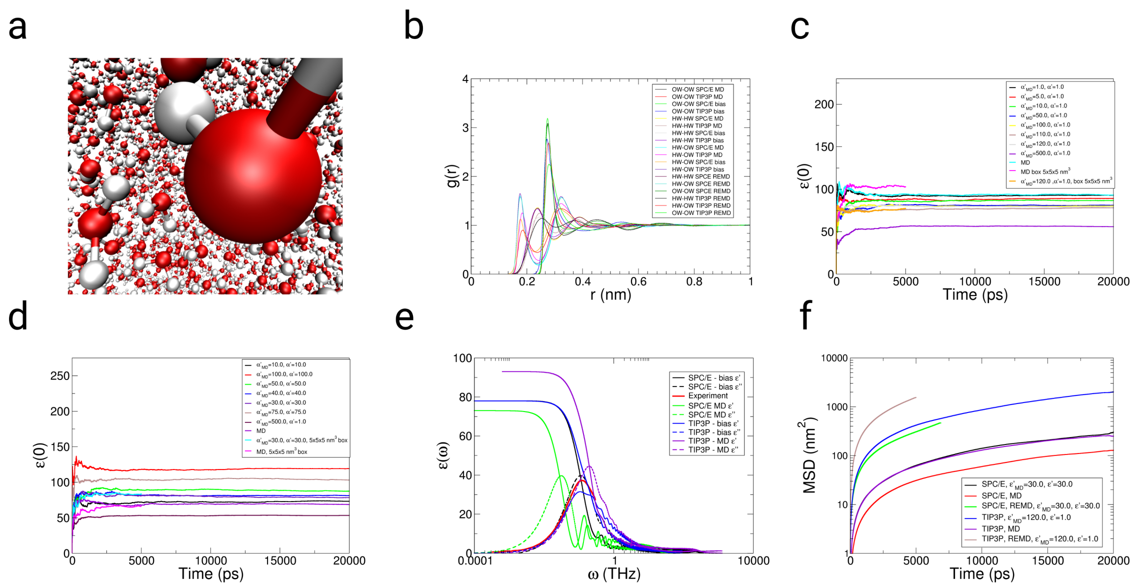

Fig. S2. Results from simulations of SPC/E and TIP3P water using MD and the path sampling technique. (a) Final configuration of a SPC/E water simulation after 20 ns of simulation. (b) Site-site radial distribution functions (RDF) (water oxygen - OW, water hydrogen - HW) from biased and unbiased MD simulations of SPC/E and TIP3P-water. (c, d) Static permittivities $\epsilon(0)$ as function of MD-simulation time using different coupling η_{MD} and η''-parameters in the individual simulations (c) - SPC/E water, (d) TIP3P water. (e) Frequency dependent permittivities $\epsilon(\omega)$ as function of frequency ω obtained from biased and un-biased simulations of SPC/E and TIP3P water (SPC/E $\eta_{MD} = \eta'$=30.0, TIP3P η_{MD}=120, η=1.0). (f) Mean-square displacement as function of simulation time from MD- and biased simulations of SPC/E and TIP3P water. Experimental values for the dielectric spectrum ($\epsilon''(\omega)$) taken from ref. [17]. For the REMD simulations, we used 4 replicas at an identical target temperature (see section Methods) in order to sample the properties of water at 300 K.

2.2.2. Structural and dynamic properties

We analyzed the site-site specific radial distribution functions (RDF) of SPC/E and TIP3P-water obtained from biased and un-biased simulations of the system with a dimension of 125 nm^3. We measured the RDFs between water-oxygen, water-oxygen (OW-OW), water-oxygen, water-hydrogen (OW-HW) and water-hydrogen, water-hydrogen (HW-HW). Our results on the structural and dynamic properties are summarized in Table 2.

In unbiased MD-simulations of SPC/E water the RDFs contain maxima ((water oxygen-oxygen)(OW-OW) 0.276 nm (1), 0.45 nm (2) and 0.69 nm (3)), ((water oxygen-hydrogen)(OW-HW) 0.176 nm (1) and 0.326 nm (2)), while biased simulations of the same system using η_{MD}=40.0 and η'=40.0 contain maxima ((water oxygen-oxygen)(OW-OW) 0.276 nm (1), 0.45 nm (2) and 0.676 nm (3)), ((water oxygen-hydrogen)(OW-HW) 0.176 nm (1) and 0.322 nm (2)). In unbiased MD-simulations of TIP3P water we find maxima ((water oxygen-oxygen)(OW-OW) 0.278 nm (1), 0.45 nm (2) and 0.698 nm (3)), ((water oxygen-hydrogen)(OW-HW) 0.184 nm (1) and 0.322 nm (2)), while biased simulations of the same system using η_{MD}=120.0 and η'=1.0 contain maxima ((water oxygen-oxygen)(OW-OW) 0.272 nm (1), 0.54 nm (2) and 0.802 nm (3)), ((water oxygen-hydrogen)(OW-HW) 0.178 nm (1) and 0.314 nm (2)) (see Figure 2S b). Using the same η-coupling parameters, the REMD simulations of both SPC/E and TIP3P lead to approximately the same results as in the sequential simulations.

The relative probability densities as the different positions of the maxima are shifted in the biased simulations relative to the unbiased MD-simulations. Between the un-biased and the biased simulation of SPC/E water, we observe shifts (OW-OW) : 0.276 (3.08483)-0.274 nm (3.19019) (un-biased, biased) $\Delta \approx 0.11$, 0.45 (1.11738)-0.45 nm (1.04048) (un-biased, biased) $\Delta \approx 0.07$, 0.69 (1.04572)-0.676

	RDF(OWOW) 1st. d(nm) (g(r))	RDF(OWOW) 2nd.	RDF(OWOW) 3rd d(nm)	RDF (OWHW) 1st.
SPC/E, MD	0.276 nm (3.08483)	0.45 nm (1.11738)	0.69 nm (1.04572)	0.176 nm (1.601)
SPC/E, η_{MD}=30.0, η'=30.0	0.274 nm (3.19019)	0.45 nm (1.04048)	0.676 nm (1.02558)	0.176 nm (1.65284)
SPC/E, η_{MD}=30.0, η'=30.0, REMD	0.276 (3.05329)	0.45 nm (1.09015)	0.69 (1.03992)	0.178 nm (1.57627)
TIP3P, MD	0.278 nm (2.68578)	0.45 nm (1.00191)	0.698 nm (1.02386)	0.184 nm (1.25325)
TIP3P, η_{MD}=120.0, η'=1.0	0.272 nm (2.76855)	0.54 nm (1.06742)	0.802 nm (1.00734)	0.178 nm (1.12709)
TIP3P, η_{MD}=120.0, η'=1.0, REMD	0.28 nm (2.25373)	0.56 nm (1.04699)	0.806 nm (1.00143)	0.186 nm (0.902549)
Experiment[18]	0.288 nm (3.09)	0.45 nm (1.14)	0.673 nm (1.07)	0.185 nm (1.38)

Table 1. Results from biased and unbiased MD-simulations of SPC/E and TIP3P water in a comparison with experimental results [17–21]. For the biased simulations, we used 20 bias replicas, a coupling strength $\beta = 5 \times 10^{-4}$, and η_{MD}=30.0 (SPC/E), 110.0 (TIP3P), η'=30.0 (SPC/E), 1.0 (TIP3P) which influences the fluctuation strength and quantities connected to the correlation behavior as the static and frequency dependent permittivity ($\epsilon(0)$, $\epsilon(\omega)$). For the REMD simulations, we used 4 replicas at the same target temperature of 300 K (see section Methods), while we used the same coupling parameters and number of bias-replicas. From left to right : Maxima of the site-site specific radial distribution functions averaged over the individual trajectories (water-oxygen, water-oxygen (OW-OW)) (position d(nm), g(r) value in brackets), (water-oxygen, water-hydrogen (OW-HW)). Self diffusion coefficients (D). Static permittivity. Maximal value of the imaginary part of the frequency dependent permittivity ($\epsilon''(\omega)_{max}$). Frequency ω at the maximal value of $\epsilon''(\omega)$. We omitted this analysis for the REMD simulations.

nm (1.02558) (un-biased, biased) $\Delta \approx 0.07$, (OW-HW) : 0.176 (1.601)-0.176 nm (1.65284) (un-biased, biased) $\Delta \approx 0.05$, and 0.326 (1.58315)-0.326 nm (1.57126) (un-biased, biased) $\Delta \approx 0.01$. In the biased and un-biased simulations of TIP3P water, we measure shifts (OW-OW) : 0.278 (2.68578)-0.272 nm (2.76855) (un-biased, biased) $\Delta \approx 0.08$, 0.45 (1.00191)-0.54 nm (1.06742) (un-biased, biased) $\Delta \approx 0.06$, 0.698 (1.02386)-0.802 nm (1.00734) (un-biased, biased) $\Delta \approx 0.02$, (OW-HW) : 0.184 (1.25325)-0.178 nm (1.12709) (un-biased, biased) $\Delta \approx 0.1$, and 0.322 (1.44738)-0.314 nm (1.35551) (un-biased, biased) $\Delta \approx 0.09$. In terms of the structural properties, the REMD simulations of both water systems (TIP3P and SPC/E) reach an approximately identical behavior as in the sequential simulations.

In the MD-simulation of SPC/E water, we measure a self-diffusion coefficient D of 1.0866 ($\pm$ 0.2011) 10^{-5} $\frac{cm^2}{s}$. The self diffusion in the enhanced sampling simulation of SPC/E water using η_{MD}=30.0 and η'=30.0, equals 2.4436 ($\pm$ 0.1665) 10^{-5} $\frac{cm^2}{s}$. In the MD-simulation of TIP3P water, we measure a self-diffusion coefficient D with a value of 2.3045 ($\pm$ 0.1547) 10^{-5} $\frac{cm^2}{s}$, while the enhanced simulation of the same system using η_{MD}=120.0 and η'=1.0 results in a value for D of 17.0078 ($\pm$ 5.2154) 10^{-5} $\frac{cm^2}{s}$ (see Figure 2S f). The self-diffusion coefficient observed in the REMD simulations exceeds the values from the sequential runs, where we observe a value of 10.3925 10^{-5} $\frac{cm^2}{s}$ for SPC/E water and a value of 50.5028 10^{-5} $\frac{cm^2}{s}$ for TIP3P water.

2.2.3. Discussion

SPC/E water and the TIP3P model have been investigated in a number of MD simulation studies [15,17,22–26]. For the self diffusion coefficients of SPC/E water, a coefficient D of 3.02 10^{-5} $\frac{cm^2}{s}$ has been determined in simulations, while simulations of TIP3P yielded 6.23 10^{-5} $\frac{cm^2}{s}$ [25]. An experimental value of 2.30 10^{-5} $\frac{cm^2}{s}$ has been reported for the self-diffusion coefficient of water [19,20]. The self-diffusion coefficient obtained in the biased simulation of SPC/E water is in very good agreement with that experimental value. Maxima in the site-site specific RDFs ((OW-OW) 0.288 nm (1), 0.45 nm (2) and 0.673 nm (3)), ((OW-HW) 0.185 nm (1) and 0.330 nm (2)) have been determined in neutron diffraction experiments [18], while site-site specific RDFs ((OW-OW) 0.278 nm (1), 0.45 nm (2) and 0.685 nm (3) (SPC/E)) ((OW-OW) 0.277 nm (1), 0.45 nm (2) and 0.684 nm (3) (TIP3P)), ((OW-HW) 0.180 nm (1) and 0.327 nm (2) (SPC/E)) ((OW-HW) 0.183 nm (1) and 0.322 nm (2) (TIP3P)) have been measured in simulations [25]. Our results from the biased and un-biased simulations of

	RDF(OWHW) 2nd. d(nm) (g(r))	D ($10^{-5} \frac{cm^2}{s}$)	$\epsilon(0)$	$\epsilon''(\omega)_{max}$	$\omega_{max}(THz)$
SPC/E, MD	0.326 nm (1.58315)	1.0866 (± 0.2011)	68.7/66.5	39.69	0.031
SPC/E, η_{MD}=30.0, η'=30.0	0.326 nm (1.57126)	2.4436 (± 0.1665)	78.4	39.7	0.11
SPC/E, η_{MD}=30.0, η'=30.0, REMD	0.328 nm (1.55756)	10.3925 (± 1.8549)	77.7	–	–
TIP3P, MD	0.322 nm (1.44738)	2.3045 (± 0.1547)	93/102	31.47	0.19
TIP3P, η_{MD}=120.0, η'=1.0	0.314 nm (1.35551)	17.0078 (± 5.2154)	78.0	44.5	0.107
TIP3P, η_{MD}=120.0, η'=1.0, REMD	0.326 nm (1.34292)	50.5028 (± 4.7595)	79.7	–	–
Experiment[17–21]	0.33 nm (1.6)	2.30	78.4	37.24	0.12

Table 2. Results from biased and unbiased MD-simulations of SPC/E and TIP3P water in a comparison with experimental results [17–21]. For the biased simulations, we used 20 bias replicas, a coupling strength $\beta = 5 \times 10^{-4}$, and η_{MD}=30.0 (SPC/E), 110.0 (TIP3P), η'=30.0 (SPC/E), 1.0 (TIP3P) which influences the fluctuation strength and quantities connected to the correlation behavior as the static and frequency dependent permittivity ($\epsilon(0)$, $\epsilon(\omega)$). For the REMD simulations, we used 4 replicas at the same target temperature of 300 K (see section Methods), while we used the same coupling parameters and number of bias-replicas. From left to right : Maxima of the site-site specific radial distribution functions averaged over the individual trajectories (water-oxygen, water-oxygen (OW-OW)) (position d(nm), g(r) value in brackets), (water-oxygen, water-hydrogen (OW-HW)). Self diffusion coefficients (D). Static permittivity. Maximal value of the imaginary part of the frequency dependent permittivity ($\epsilon''(\omega)_{max}$). Frequency ω at the maximal value of $\epsilon''(\omega)$. We omitted this analysis for the REMD simulations.

SPC/E-water are in good agreement with their results. Investigations on the dielectric properties of water are of specific interest and especially the SPC/E water model has been found to describe frequency dependent dielectric permittivities shifted by -0.1 THz to the experimental maximum of $\epsilon''(\omega)$ [17], while a value of 70.7 [23] for the static permittivity value $\epsilon(0)$ has been reported. That value is shifted in comparison to the experiment by a value of approximately 8, while the experimental value is at 78.4 at room temperature [21]. In contrast to the SPC/E water model, static permittivities in the range from 93 to 104 have been observed for the TIP3P water model, while its peak for ϵ'' in the dielectric spectrum is shifted by approximately +0.1 THz in relation to the experimental value [17,24]. In terms of the static and frequency dependent permittivity value, our result from the biased simulation of SPC/E water is in good agreement with the experiment. That also holds for the REMD simulation in the case of the static permittivity, while we omitted the frequency dependent analysis due to the dependency of the dynamics of the system from the exchange frequency. The dielectric behavior of water plays an important role in the behavior of biomolecules, while static dielectric constants for biomolecular systems ranging from 10 to 20 have been reported [27,28]. Especially the non-polar region of proteins, which is shielded from the solvent in its folded state, can contain values for $\epsilon(0)$ of approximately 5 [29]. However, here it remains questionable to speak of a dielectric constant, which is a macroscopic quantity and might not be reducable to a range below the nm length-scale. Recent studies investigated the effect of ions and the folding of alanine-pentapeptide on the dielectric relaxation behavior of the underlying systems [30,31].

We conclude that the presented algorithm can change the time-dependent properties of SPC/E and TIP3P water through a scaling of η_{MD} and η', as we stated in the main body of the text in the Section : Methods. Especially the parameter η_{MD} can modify the time-dependent decay of the total dipole moment in comparison with the conventional MD propagation. We found that the magnitude η' affects the fluctuations of the applied bias averaged and also can change with the number of parallel replicas, as we find in the REMD simulations. For the TIP3P model, the charge density has a high value that a large value for η_{MD}=120.0 has to be used to scale down the static permittivity to a value of 78. That scaling has a significant effect on the water structure in comparison with the experimental result and shifts the location of maxima in the RDF. In the case of the SPC/E water, a scaling of η' to larger values yields the correct static permittivity value, an approximately correct water-structure in comparison with the experiment as well as a comparatively good agreement in its dielectric relaxation

behavior with experimental results. As we find in the REMD case, we observe that the combination of REMD with the multiple path-sampling can sample the structural properties of water in terms of the radial distribution function and the static permittivities in the same way as the sequential technique.

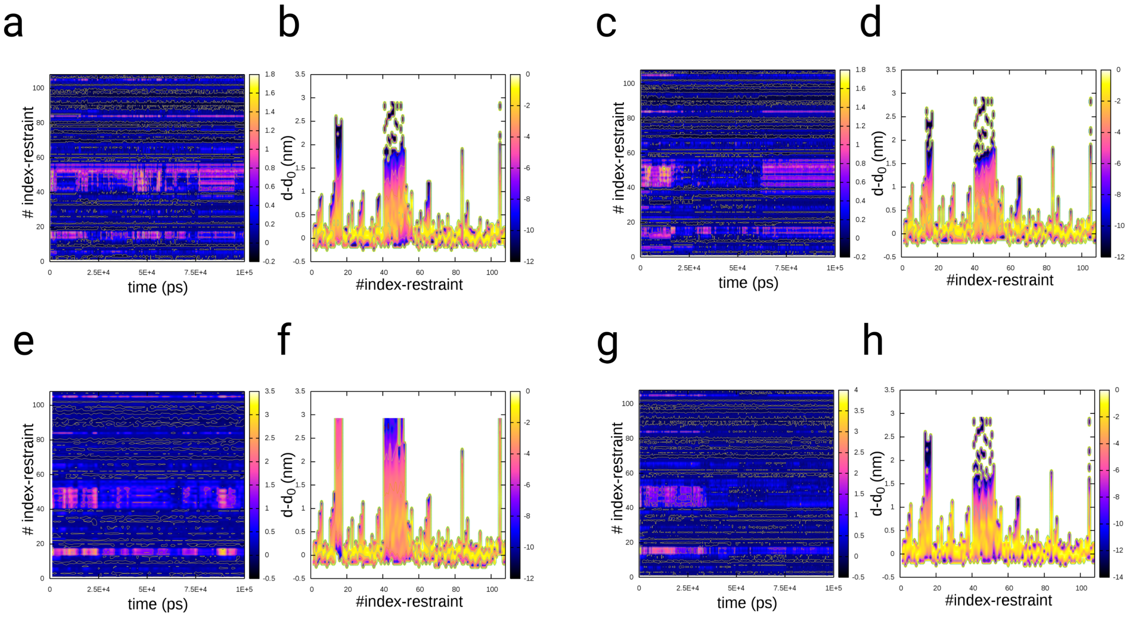

Fig. S3. (a, c, e, g) Difference $d_{ij}(t) - d_{ij}(exp)$ of the distance between NMR-distance restraint indexed atoms ij and the experimental value as function of time and the restraint index. (b, d, f, g) Probability of finding the system within the difference of the distance between NMR-distance restraint indexed atoms ij and the experimental value $d_{ij}(t) - d_{ij}(exp)$. (a, b) Results from un-restrained path-sampling simulation in implicit solvent. (c, d) Results from restrained path-sampling simulation in implicit solvent using NMR-restraints. (e, f) Results from un-restrained path-sampling simulation in explicit solvent. (g, h) Results from restrained path-sampling simulation in explicit solvent using NMR-restraints.

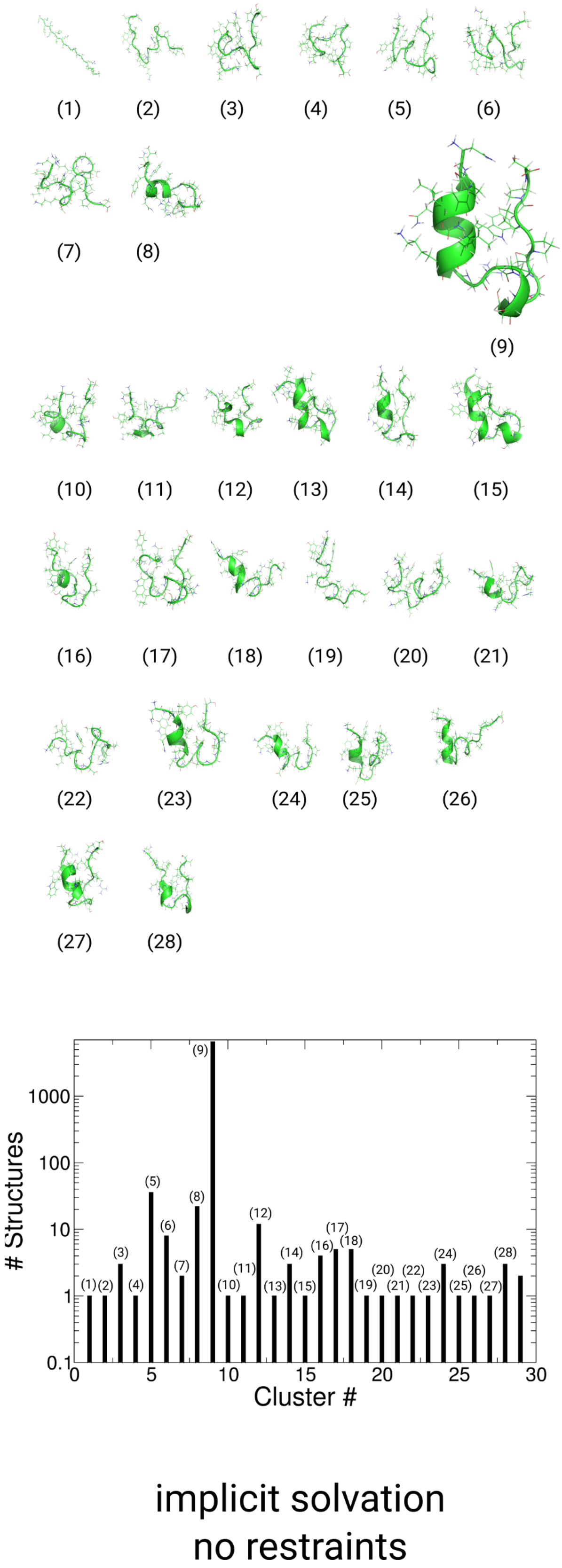

Fig. S4. Cluster populations from the cluster analysis of the simulations in implicit solvent (unrestrained).

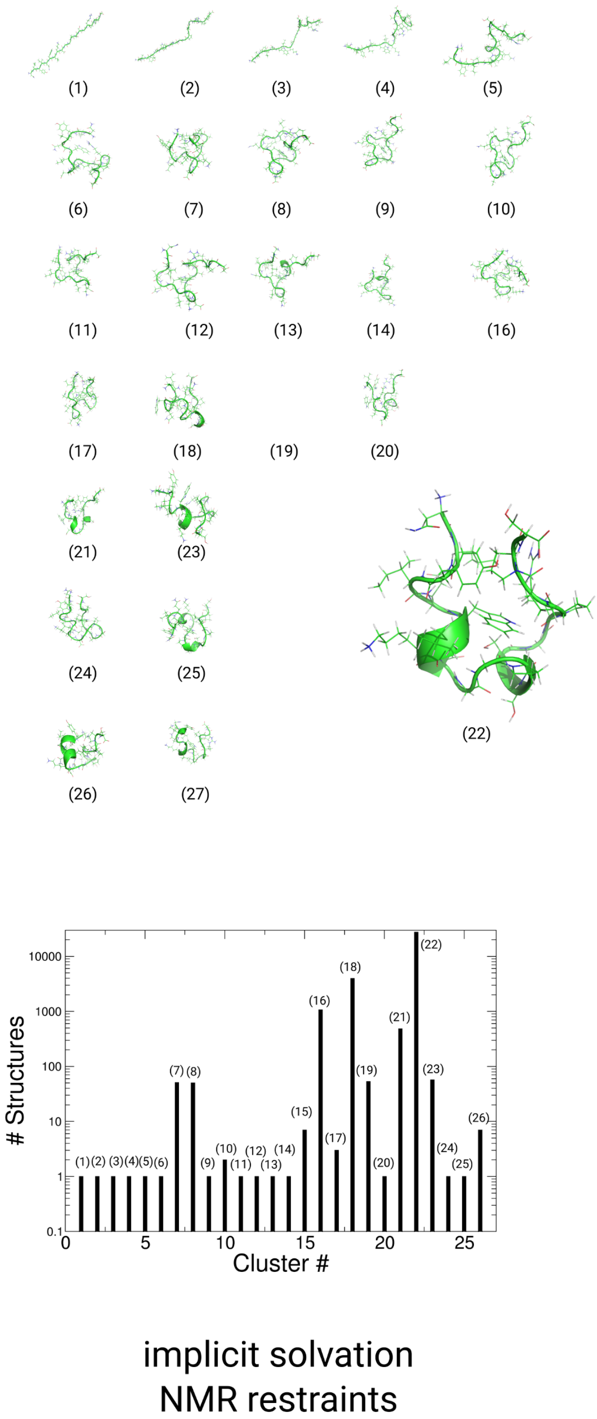

Fig. S5. Cluster populations from the cluster analysis of the simulations in implicit solvent (NMR restraints).

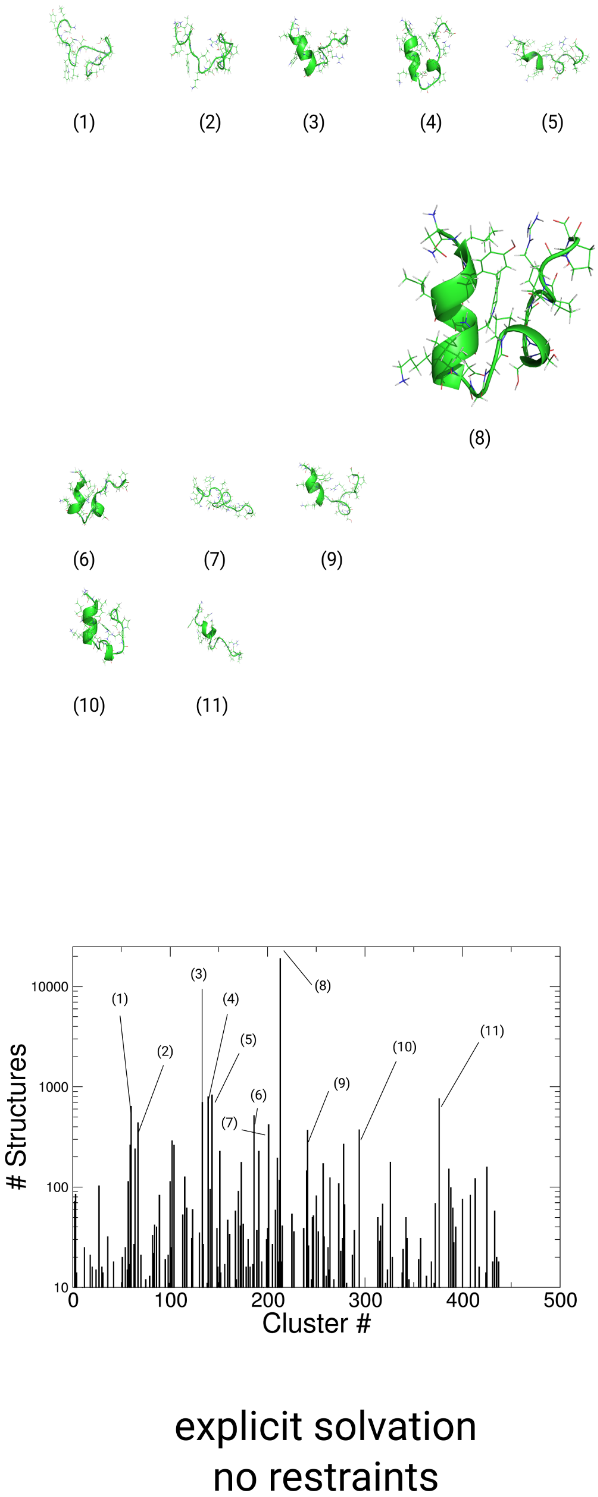

Fig. S6. Cluster populations from the cluster analysis of the simulations in explicit solvent (unrestrained).

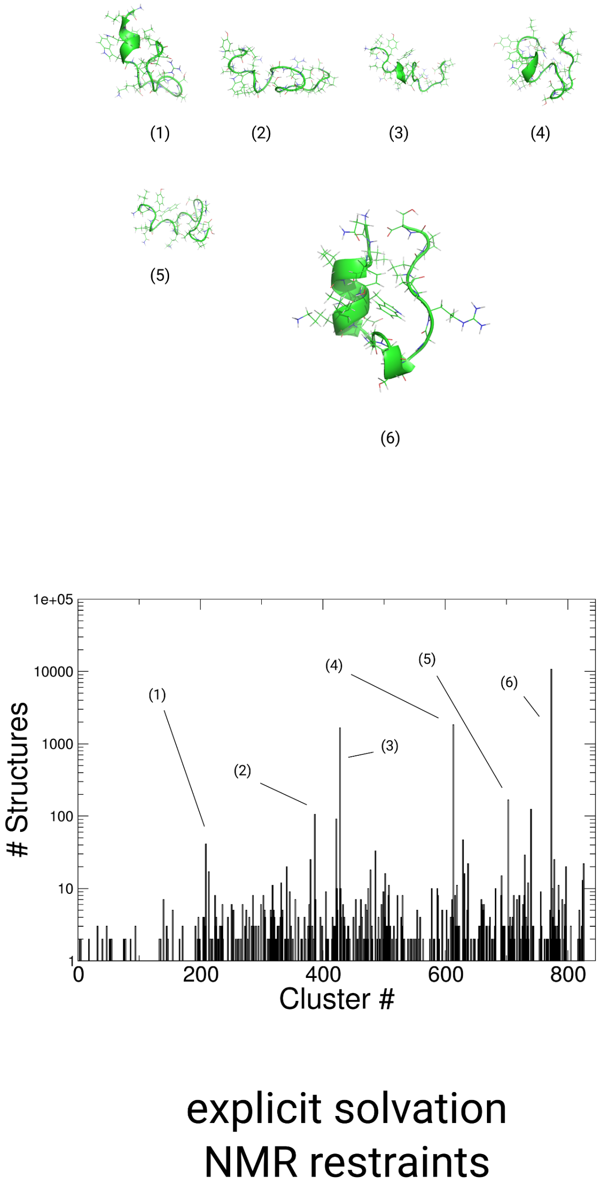

Fig. S7. Cluster populations from the cluster analysis of the simulations in explicit solvent (NMR restraints).

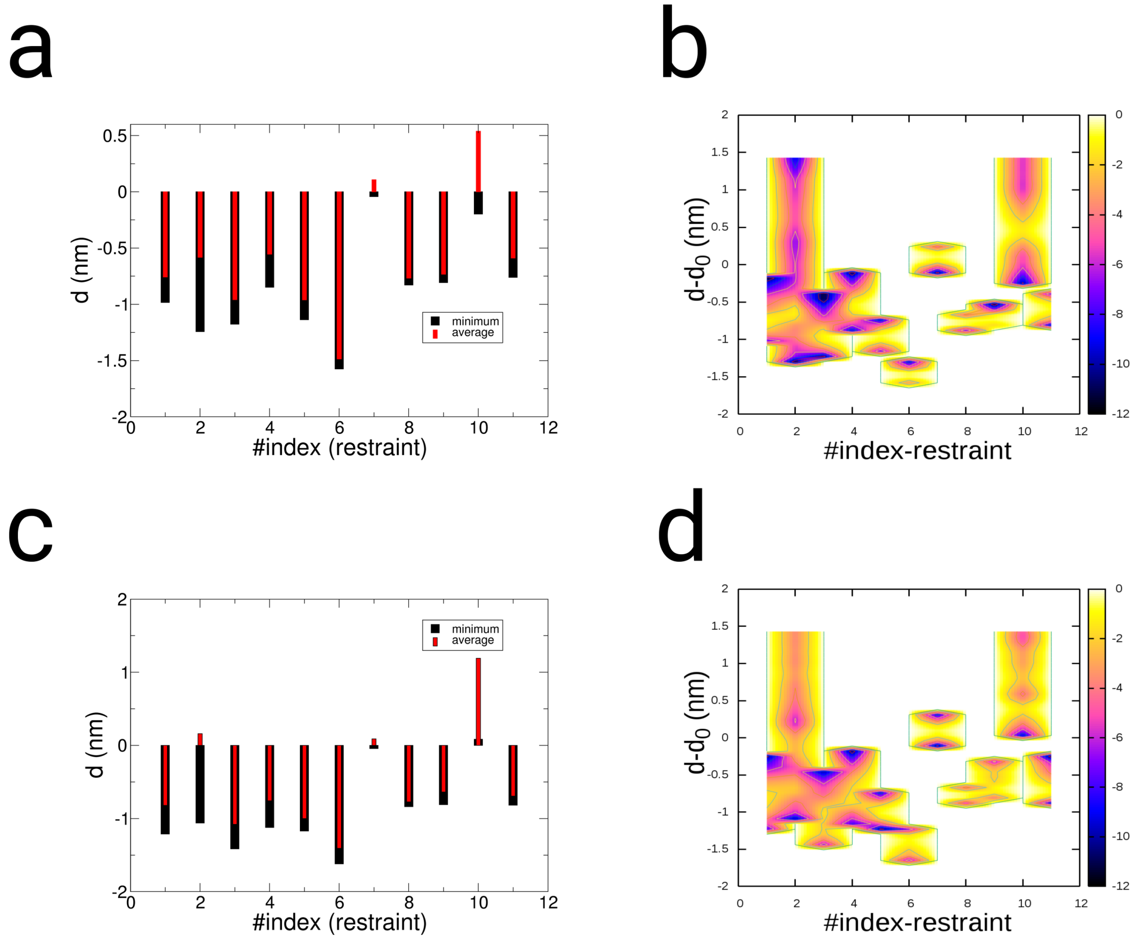

Fig. S8. (a, c) Average and minimal distance values along 11 distance restraints. (b, d) Population densities of each of the 11 distance restraints as function of the difference between the distance and the chemical restraint distance value as given in ref. [32]. (a, b) Resuls from restrained simulations in implicit solvent. (c, d) Results from restrained simulations in explicit solvent.

3. Restraint parameters

- Experimental NMR-NOESY restraint data used for the restraint simulations of the TrpCage miniprotein from ref. [33] (restraint-index, residue number 1, atom-name, residue number 2, atom-name, distance (Å)):

1	2LEU	H	1ASN	HA	2.500
2	1ASN	HB2	2LEU	H	2.900
3	3TYR	H	2LEU	H	2.500
4	4ILE	H	2LEU	H	3.300
5	5GLN	H	2LEU	HA	2.500
6	5GLN	HB2	2LEU	HA	2.900
7	2LEU	HA	3TYR	H	2.900
8	2LEU	HB2	3TYR	H	2.900
9	4ILE	H	3TYR	H	2.000
10	3TYR	HB2	3TYR	HA	2.000
11	6TRP	H	3TYR	HA	2.900
12	6TRP	HB2	3TYR	HA	2.900
13	6TRP	HE3	3TYR	HA	2.900
14	19PRO	HB2	3TYR	HA	3.300
15	19PRO	HD2	3TYR	HA	2.900
16	19PRO	HG2	3TYR	HA	2.900
17	19PRO	HG2	3TYR	HB2	2.900
18	3TYR	HA	4ILE	H	2.900
19	4ILE	HB	4ILE	H	2.000
20	5GLN	H	4ILE	H	2.500
21	6TRP	H	4ILE	H	3.300
22	4ILE	HB	4ILE	HA	2.900
23	7LEU	H	4ILE	HA	2.500
24	4ILE	HA	5GLN	H	2.900
25	4ILE	HB	5GLN	H	2.500
26	5GLN	HB2	5GLN	H	2.000
27	7LEU	H	5GLN	H	3.300
28	8LYS	H	5GLN	HA	2.500
29	8LYS	HB2	5GLN	HA	2.900
30	6TRP	H	5GLN	HB2	2.900
31	5GLN	HA	6TRP	H	2.900
32	5GLN	HA	6TRP	H	2.900
33	6TRP	HB2	6TRP	H	2.000
34	6TRP	HE3	6TRP	H	3.300
35	7LEU	H	6TRP	H	2.500
36	6TRP	HD1	6TRP	HA	2.500
37	9ASP	H	6TRP	HA	3.300
38	9ASP	HB2	6TRP	HA	3.300
39	6TRP	HA	6TRP	HB2	2.000
40	6TRP	HD1	6TRP	HB2	2.500
41	16ARG	HB2	6TRP	HD1	2.900
42	16ARG	HD2	6TRP	HD1	2.900
43	16ARG	HG2	6TRP	HD1	2.900
44	18PRO	HA	6TRP	HD1	3.300
45	16ARG	H	6TRP	HE1	3.300

46	16ARG	HB2	6TRP	HE1	2.900
47	17PRO	HA	6TRP	HE1	2.900
48	18PRO	HA	6TRP	HE1	2.900
49	12PRO	HA	6TRP	HZ2	2.000
50	17PRO	HA	6TRP	HZ2	3.300
51	18PRO	HD2	6TRP	HZ2	3.300
52	12PRO	HG2	6TRP	HH2	2.900
53	7LEU	HG	6TRP	HZ3	3.300
54	7LEU	HA	6TRP	HE3	2.900
55	7LEU	H	6TRP	HE3	2.500
56	7LEU	HG	6TRP	HE3	2.000
57	6TRP	HA	7LEU	H	2.900
58	7LEU	HB2	7LEU	H	2.000
59	7LEU	HG	7LEU	H	2.500
60	8LYS	H	7LEU	H	2.500
61	9ASP	H	7LEU	H	3.300
62	7LEU	HB2	7LEU	HA	2.900
63	9ASP	H	7LEU	HA	3.300
64	10GLY	H	7LEU	HA	3.300
65	11GLY	H	7LEU	HA	2.000
66	11GLY	HA2	7LEU	HA	2.900
67	7LEU	HA	8LYS	H	2.900
68	7LEU	HB2	8LYS	H	2.500
69	8LYS	HB2	8LYS	H	2.000
70	9ASP	H	8LYS	H	2.000
71	10GLY	H	8LYS	H	3.300
72	8LYS	HA	9ASP	H	2.900
73	8LYS	HB2	9ASP	H	2.900
74	9ASP	HB2	9ASP	H	2.000
75	10GLY	H	9ASP	H	2.000
76	11GLY	H	9ASP	H	3.300
77	9ASP	HA	10GLY	H	2.900
78	11GLY	H	10GLY	H	2.000
79	10GLY	HA2	11GLY	H	2.900
80	11GLY	HA2	11GLY	H	2.000
81	12PRO	HB2	12PRO	HD2	2.500
82	13SER	H	12PRO	HD2	2.900
83	13SER	H	12PRO	HB2	3.300
84	6TRP	HH2	12PRO	HA	3.300
85	13SER	H	12PRO	HA	2.900
86	14SER	H	12PRO	HA	3.300
87	15GLY	H	12PRO	HA	3.300
88	13SER	HB2	13SER	H	2.500
89	14SER	H	13SER	H	2.000
90	13SER	HA	14SER	H	2.900
91	15GLY	H	14SER	H	2.000
92	14SER	H	14SER	HB2	2.000
93	15GLY	H	14SER	HB2	3.300
94	13SER	HA	15GLY	H	2.900
95	14SER	HA	15GLY	H	2.900

96	15GLY	HA2	15GLY	H	2.000
97	15GLY	HA2	16ARG	H	2.900
98	15GLY	H	16ARG	H	2.000
99	17PRO	HD2	16ARG	HA	2.000
100	17PRO	HD2	16ARG	HB2	2.500
101	17PRO	HD2	17PRO	HB2	2.900
102	18PRO	HD2	17PRO	HA	2.500
103	18PRO	HD2	18PRO	HB2	2.900
104	19PRO	HD2	18PRO	HB2	2.500
105	6TRP	HD1	19PRO	HD2	3.300
106	18PRO	HA	19PRO	HD2	2.500
107	19PRO	HD2	19PRO	HB2	2.900
108	20SER	H	19PRO	HB2	2.900

- Experimental chemical crosslinking restraint data used for the restraint simulations of the Killer Cell Lectin-like Receptor Subfamily B Member 1A from ref. [32] (restraint-index, residue number 1, atom- name, residue number 2, atom-name, distance (Å)):

1	166LYS	N	179LYS	N	20.000
2	179LYS	N	196LYS	N	20.000
3	148LYS	N	196LYS	N	20.000
4	146LYS	N	196LYS	N	20.000
5	125LYS	N	212LYS	N	20.000
6	146LYS	N	148LYS	N	20.000
7	147GLU	N	148LYS	N	12.000
8	121ASP	N	125LYS	N	12.000
9	123ASP	N	125LYS	N	12.000
10	143ASP	N	146LYS	N	12.000
11	176ASP	N	196LYS	N	12.000
12	176ASP	N	179LYS	N	12.000

- Restraint data set used for the restraint simulations of the Killer Cell Lectin-like Receptor Subfamily B Member 1A from ref. [32] (restraint-index, residue number 1, atom-name, residue number 2, atom-name, distance (Å)). We note that we considered each distance-value to be significantly lower than the chemical cross-linking distances as described before (7.5 Å). The distance value only affects the sign of the overlappling segment of the path-increment dL in contrast to harmonic potentials:

1	212LYS	N	121ASP	N	7.500
2	212LYS	N	121ASP	H	7.500
3	212LYS	N	121ASP	CA	7.500
4	212LYS	N	121ASP	HA	7.500
5	212LYS	N	121ASP	CB	7.500
6	212LYS	N	121ASP	HB1	7.500
7	212LYS	N	121ASP	HB2	7.500
8	212LYS	N	121ASP	CG	7.500
9	212LYS	N	121ASP	OD1	7.500
10	212LYS	N	121ASP	OD2	7.500
11	212LYS	N	121ASP	C	7.500
12	212LYS	N	121ASP	O	7.500

13	125LYS	N	123ASP	N	7.500
14	125LYS	N	123ASP	H	7.500
15	125LYS	N	123ASP	CA	7.500
16	125LYS	N	123ASP	HA	7.500
17	125LYS	N	123ASP	CB	7.500
18	125LYS	N	123ASP	HB1	7.500
19	125LYS	N	123ASP	HB2	7.500
20	125LYS	N	123ASP	CG	7.500
21	125LYS	N	123ASP	OD1	7.500
22	125LYS	N	123ASP	OD2	7.500
23	125LYS	N	123ASP	C	7.500
24	125LYS	N	123ASP	O	7.500
25	212LYS	N	125LYS	N	7.500
26	212LYS	N	125LYS	H	7.500
27	212LYS	N	125LYS	CA	7.500
28	212LYS	N	125LYS	HA	7.500
29	212LYS	N	125LYS	CB	7.500
30	212LYS	N	125LYS	HB1	7.500
31	212LYS	N	125LYS	HB2	7.500
32	212LYS	N	125LYS	CG	7.500
33	212LYS	N	125LYS	HG1	7.500
34	212LYS	N	125LYS	HG2	7.500
35	212LYS	N	125LYS	CD	7.500
36	212LYS	N	125LYS	HD1	7.500
37	212LYS	N	125LYS	HD2	7.500
38	212LYS	N	125LYS	CE	7.500
39	212LYS	N	125LYS	HE1	7.500
40	212LYS	N	125LYS	HE2	7.500
41	212LYS	N	125LYS	NZ	7.500
42	212LYS	N	125LYS	HZ1	7.500
43	212LYS	N	125LYS	HZ2	7.500
44	212LYS	N	125LYS	HZ3	7.500
45	212LYS	N	125LYS	C	7.500
46	212LYS	N	125LYS	O	7.500
47	146LYS	N	143ASP	N	7.500
48	146LYS	N	143ASP	H	7.500
49	146LYS	N	143ASP	CA	7.500
50	146LYS	N	143ASP	HA	7.500
51	146LYS	N	143ASP	CB	7.500
52	146LYS	N	143ASP	HB1	7.500
53	146LYS	N	143ASP	HB2	7.500
54	146LYS	N	143ASP	CG	7.500
55	146LYS	N	143ASP	OD1	7.500
56	146LYS	N	143ASP	OD2	7.500
57	146LYS	N	143ASP	C	7.500
58	146LYS	N	143ASP	O	7.500
59	196LYS	N	146LYS	N	7.500
60	148LYS	N	146LYS	N	7.500
61	196LYS	N	146LYS	H	7.500
62	148LYS	N	146LYS	H	7.500

63	196LYS	N	146LYS	CA	7.500
64	148LYS	N	146LYS	CA	7.500
65	196LYS	N	146LYS	HA	7.500
66	148LYS	N	146LYS	HA	7.500
67	196LYS	N	146LYS	CB	7.500
68	148LYS	N	146LYS	CB	7.500
69	196LYS	N	146LYS	HB1	7.500
70	148LYS	N	146LYS	HB1	7.500
71	196LYS	N	146LYS	HB2	7.500
72	148LYS	N	146LYS	HB2	7.500
73	196LYS	N	146LYS	CG	7.500
74	148LYS	N	146LYS	CG	7.500
75	196LYS	N	146LYS	HG1	7.500
76	148LYS	N	146LYS	HG1	7.500
77	196LYS	N	146LYS	HG2	7.500
78	148LYS	N	146LYS	HG2	7.500
79	196LYS	N	146LYS	CD	7.500
80	148LYS	N	146LYS	CD	7.500
81	196LYS	N	146LYS	HD1	7.500
82	148LYS	N	146LYS	HD1	7.500
83	196LYS	N	146LYS	HD2	7.500
84	148LYS	N	146LYS	HD2	7.500
85	196LYS	N	146LYS	CE	7.500
86	148LYS	N	146LYS	CE	7.500
87	196LYS	N	146LYS	HE1	7.500
88	148LYS	N	146LYS	HE1	7.500
89	196LYS	N	146LYS	HE2	7.500
90	148LYS	N	146LYS	HE2	7.500
91	196LYS	N	146LYS	NZ	7.500
92	148LYS	N	146LYS	NZ	7.500
93	196LYS	N	146LYS	HZ1	7.500
94	148LYS	N	146LYS	HZ1	7.500
95	196LYS	N	146LYS	HZ2	7.500
96	148LYS	N	146LYS	HZ2	7.500
97	196LYS	N	146LYS	HZ3	7.500
98	148LYS	N	146LYS	HZ3	7.500
99	196LYS	N	146LYS	C	7.500
100	148LYS	N	146LYS	C	7.500
101	196LYS	N	146LYS	O	7.500
102	148LYS	N	146LYS	O	7.500
103	148LYS	N	147GLU	N	7.500
104	148LYS	N	147GLU	H	7.500
105	148LYS	N	147GLU	CA	7.500
106	148LYS	N	147GLU	HA	7.500
107	148LYS	N	147GLU	CB	7.500
108	148LYS	N	147GLU	HB1	7.500
109	148LYS	N	147GLU	HB2	7.500
110	148LYS	N	147GLU	CG	7.500
111	148LYS	N	147GLU	HG1	7.500
112	148LYS	N	147GLU	HG2	7.500

113	148LYS	N	147GLU	CD	7.500
114	148LYS	N	147GLU	OE1	7.500
115	148LYS	N	147GLU	OE2	7.500
116	148LYS	N	147GLU	C	7.500
117	148LYS	N	147GLU	O	7.500
118	196LYS	N	148LYS	N	7.500
119	196LYS	N	148LYS	H	7.500
120	196LYS	N	148LYS	CA	7.500
121	196LYS	N	148LYS	HA	7.500
122	196LYS	N	148LYS	CB	7.500
123	196LYS	N	148LYS	HB1	7.500
124	196LYS	N	148LYS	HB2	7.500
125	196LYS	N	148LYS	CG	7.500
126	196LYS	N	148LYS	HG1	7.500
127	196LYS	N	148LYS	HG2	7.500
128	196LYS	N	148LYS	CD	7.500
129	196LYS	N	148LYS	HD1	7.500
130	196LYS	N	148LYS	HD2	7.500
131	196LYS	N	148LYS	CE	7.500
132	196LYS	N	148LYS	HE1	7.500
133	196LYS	N	148LYS	HE2	7.500
134	196LYS	N	148LYS	NZ	7.500
135	196LYS	N	148LYS	HZ1	7.500
136	196LYS	N	148LYS	HZ2	7.500
137	196LYS	N	148LYS	HZ3	7.500
138	196LYS	N	148LYS	C	7.500
139	196LYS	N	148LYS	O	7.500
140	179LYS	N	166LYS	N	7.500
141	179LYS	N	166LYS	H	7.500
142	179LYS	N	166LYS	CA	7.500
143	179LYS	N	166LYS	HA	7.500
144	179LYS	N	166LYS	CB	7.500
145	179LYS	N	166LYS	HB1	7.500
146	179LYS	N	166LYS	HB2	7.500
147	179LYS	N	166LYS	CG	7.500
148	179LYS	N	166LYS	HG1	7.500
149	179LYS	N	166LYS	HG2	7.500
150	179LYS	N	166LYS	CD	7.500
151	179LYS	N	166LYS	HD1	7.500
152	179LYS	N	166LYS	HD2	7.500
153	179LYS	N	166LYS	CE	7.500
154	179LYS	N	166LYS	HE1	7.500
155	179LYS	N	166LYS	HE2	7.500
156	179LYS	N	166LYS	NZ	7.500
157	179LYS	N	166LYS	HZ1	7.500
158	179LYS	N	166LYS	HZ2	7.500
159	179LYS	N	166LYS	HZ3	7.500
160	179LYS	N	166LYS	C	7.500
161	179LYS	N	166LYS	O	7.500
162	196LYS	N	176ASP	N	7.500

163	179LYS	N	176ASP	N	7.500
164	196LYS	N	176ASP	H	7.500
165	179LYS	N	176ASP	H	7.500
166	196LYS	N	176ASP	CA	7.500
167	179LYS	N	176ASP	CA	7.500
168	196LYS	N	176ASP	HA	7.500
169	179LYS	N	176ASP	HA	7.500
170	196LYS	N	176ASP	CB	7.500
171	179LYS	N	176ASP	CB	7.500
172	196LYS	N	176ASP	HB1	7.500
173	179LYS	N	176ASP	HB1	7.500
174	196LYS	N	176ASP	HB2	7.500
175	179LYS	N	176ASP	HB2	7.500
176	196LYS	N	176ASP	CG	7.500
177	179LYS	N	176ASP	CG	7.500
178	196LYS	N	176ASP	OD1	7.500
179	179LYS	N	176ASP	OD1	7.500
180	196LYS	N	176ASP	OD2	7.500
181	179LYS	N	176ASP	OD2	7.500
182	196LYS	N	176ASP	C	7.500
183	179LYS	N	176ASP	C	7.500
184	196LYS	N	176ASP	O	7.500
185	179LYS	N	176ASP	O	7.500
186	196LYS	N	179LYS	N	7.500
187	196LYS	N	179LYS	H	7.500
188	196LYS	N	179LYS	CA	7.500
189	196LYS	N	179LYS	HA	7.500
190	196LYS	N	179LYS	CB	7.500
191	196LYS	N	179LYS	HB1	7.500
192	196LYS	N	179LYS	HB2	7.500
193	196LYS	N	179LYS	CG	7.500
194	196LYS	N	179LYS	HG1	7.500
195	196LYS	N	179LYS	HG2	7.500
196	196LYS	N	179LYS	CD	7.500
197	196LYS	N	179LYS	HD1	7.500
198	196LYS	N	179LYS	HD2	7.500
199	196LYS	N	179LYS	CE	7.500
200	196LYS	N	179LYS	HE1	7.500
201	196LYS	N	179LYS	HE2	7.500
202	196LYS	N	179LYS	NZ	7.500
203	196LYS	N	179LYS	HZ1	7.500
204	196LYS	N	179LYS	HZ2	7.500
205	196LYS	N	179LYS	HZ3	7.500
206	196LYS	N	179LYS	C	7.500
207	196LYS	N	179LYS	O	7.500

References

1. Torrie, G.M.; Valleau, J.P. Nonphysical sampling distributions in Monte Carlo free-energy estimation—Umbrella sampling. *J. Comput. Phys.* **1977**, *23*, 187–199.

2. Laio, A.; Parrinello, M. Escaping free-energy minima. *Proc. Natl. Acad. Sci. USA* **2002**, *99*, 12562–12566.
3. Grubmüller, H. Predicting slow structural transitions in macromolecular systems: Conformational flooding. *Phys. Rev. E* **1995**, *52*, 2893.
4. Huber, T.; Torda, A.E.; van Gunsteren, W.F. Local elevation: A method for improving the searching properties of molecular dynamics simulation. *J. Comput. Aided Mol. Des.* **1994**, *8*, 695–708.
5. Peter, E.K.; Shea, J.E. An adaptive bias-hybrid MD/kMC algorithm for protein folding and aggregation. *Phys. Chem. Chem. Phys.* **2017**, *19*, 17373–17382.
6. Peter, E.K.; Černý, J. Enriched Conformational Sampling of DNA and Proteins with a Hybrid Hamiltonian Derived from the Protein Data Bank. *Int. J. Mol. Sci.* **2018**, *19*, E3405.
7. Kleinert, H. *Path Integrals in Quantum Mechanics, Statistics, Polymer Physics, and Financial Markets*, 5th ed.; World Scientific: Singapore, 2009; pp. 1–1547.
8. Feynman, R.; Hibbs, A.R. *Quantum Mechanics and Path Integrals*; MacGraw Hill Companies: New York, NY, USA, 1965.
9. Barducci, A.; Bonomi, M.; Parrinello, M. Linking well-tempered metadynamics simulations with experiments. *Biophys. J.* **2010**, *98*, L44–L46.
10. Bolhuis, P.G.; Dellago, C.; Chandler, D. Reaction coordinates of biomolecular isomerization. *Proc. Natl. Acad. Sci. USA* **2000**, *97*, 5877.
11. Peter, E.K. Adaptive enhanced sampling with a path-variable for the simulation of protein folding and aggregation. *J. Chem. Phys.* **2017**, *147*, 214902.
12. Barducci, A.; Bussi, G.; Parrinello, M. Well-Tempered Metadynamics: A Smoothly Converging and Tunable Free-Energy Method. *Phys. Rev. Lett.* **2008**, *100*, 020603.
13. Balsera, M.A.; Wriggers, W.; Ono, Y.; Schulten, K. Principal Component Analysis and Long Time Protein Dynamics. *J. Phys. Chem.* **1996**, *100*, 2567–2572.
14. Allen, M.; Tildesley, D. *Computer Simulation of Liquids*; Clarendon Pr: Oxford, UK, 1987.
15. v. d. Spoel, D.; v. Maaren, P.J.; Berendsen, H.J.C. A systematic study of water models for molecular simulation: Derivation of water models optimized for use with a reaction field. *J. Chem. Phys.* **1998**, *108*, 10220.
16. Neumann, M.; Steinhauser, O.; Pawley, G.S. Consistent calculation of the static and frequency-dependent dielectric constant in computer simulations. *Mol. Phys.* **1984**, *52*, 97.
17. Braun, D.; Boresch, S.; Steinhauser, O. Transport and dielectric properties of water and the influence of coarse-graining: Comparing BMW, SPC/E, and TIP3P models. *J. Chem. Phys.* **2014**, *140*, 064107.
18. Soper, A.K.; Phillips, M.G. A new determination of the structure of water at 25 °C. *Chem. Phys.* **1986**, *107*, 47.
19. Mills, R. Self-diffusion in normal and heavy water in the range 1-45.deg. *J. Phys. Chem.* **1973**, *77*, 685.
20. Price, W.S.; Ide, H.; Arata, Y. Self-Diffusion of Supercooled Water to 238 K Using PGSE NMR Diffusion Measurements. *J. Phys. Chem. A* **1999**, *103*, 448.
21. Owen, B.B.; Miller, R.C.; Milner, C.E.; Cogan, H.L. The dielectric constant of water as a function of temperature and pressure. *J. Phys. Chem.* **1961**, *65*, 2065–2070.
22. Zielkiewicz, J. Structural properties of water: Comparison of the SPC, SPCE, TIP4P, and TIP5P models of water *J. Chem. Phys.* **2005**, *123*, 104501.
23. Reddy, R.R.; Berkowitz, M. The dielectric constant of SPC/E water. *Chem. Phys. Lett.* **1989**, *155*, 173–176.
24. Hess, B.; Holm, C.; v. d. Vegt, N. Osmotic coefficients of atomistic NaCl (aq) force fields. *J. Chem. Phys.* **2006**, *124*, 164509.
25. Mark, P.; Nilsson, L. Structure and Dynamics of the TIP3P, SPC, and SPC/E Water Models at 298 K *J. Phys. Chem. A* **2001**, *105*, 9954.
26. Raabe, G.; Sadus, R.J. Molecular dynamics simulation of the dielectric constant of water: The effect of bond flexibility. *J. Chem. Phys.* **2011**, *134*, 234501.
27. Pitera, J.W.; Falta, M.; van Gunsteren, W.F. Dielectric properties of proteins from simulation: the effects of solvent, ligands, pH, and temperature. *Biophys. J.* **2001**, *80*, 2546–2555.
28. Löffler, G.; Schreiber, H.; Steinhauser, O. Calculation of the dielectric properties of a protein and its solvent: theory and a case study. *J. Mol. Biol.* **1997**, *270*, 520–534.
29. Li, L.; Li, C.; Zhang, Z.; Alexov, E. On the Dielectric "Constant" of Proteins: Smooth Dielectric Function for Macromolecular Modeling and Its Implementation in DelPhi. *J. Chem. Theor. Comput.* **2013**, *9*, 2126–2136.
30. Rinne, K.F.; Geckle, S.; Netz, R. Ion-Specific Solvation Water Dynamics: Single Water versus Collective Water Effects. *J. Phys. Chem. A* **2014**, *118*, 11667–11677.

31. Rinne, K.F.; Schulz, J.C.F.; Netz, R. Impact of secondary structure and hydration water on the dielectric spectrum of poly-alanine and possible relation to the debate on slaved versus slaving water. *J. Chem. Phys.* **2015**, *142*, 215104.
32. Rozbeský, D.; Man, P.; Kavan, D.; Chmelík, J.; Černý, J.; Bezouška, K.; Novák, P. Chemical cross-linking and H/D exchange for fast refinement of protein crystal structure. *Anal. Chem.* **2012**, *84*, 867–870.
33. Neidigh, J.W.; Fesinmeyer, R.M. Designing a 20-residue protein. *Nat. Struct. Biol.* **2002**, *9*, 425–430.

© 2019 by the authors. Licensee MDPI, Basel, Switzerland. This article is an open access article distributed under the terms and conditions of the Creative Commons Attribution (CC BY) license (http://creativecommons.org/licenses/by/4.0/).
